# A novel deep transformer based CvT model for sign language recognition in visual communication

**DOI:** 10.1038/s41598-025-31558-1

**Published:** 2025-12-21

**Authors:** Jing Hao, Hezhe Pan

**Affiliations:** 1 School of Design, Zhoukou Normal University, Zhoukou, 466001 Henan China; 2 Art Design College, Loudi Vocational and Technical College, Loudi, 417000 Hunan China

**Keywords:** Artificial intelligence, Computer vision, Deep learning, Sign language, Vision transformers, Classification and taxonomy, Computational models, Image processing

## Abstract

Sign language serves as a crucial mode of communication for the deaf and hard-of-hearing communities, enabling effective interaction in daily life. With the growing advancements in Artificial Intelligence (AI) and computer vision, there has been a significant shift toward automating SLR, making communication more accessible and inclusive. Traditional AI-based approaches, such as rule-based and statistical models, struggle to handle complex hand gestures, varying lighting conditions, and occlusions. Deep learning-based methods, particularly Convolutional Neural Networks (CNNs), have improved recognition capabilities, but they often fail to capture intricate spatial and temporal dependencies that are essential for accurate classification. To address these limitations, vision transformers (ViTs) have emerged as a breakthrough technology, offering superior feature extraction through self-attention mechanisms. Unlike conventional CNNs, ViTs efficiently model long-range dependencies, enabling robust sign recognition. This study proposes a Convolutional Vision Transformer (CvT)-based model that integrates hierarchical convolutional tokenization with transformer-based attention mechanisms, optimizing both local and global feature extraction. The proposed CvT model was evaluated on a publicly available sign language digits dataset, consisting of 1,712 images across 10 different classes along with alphabet and symbol dataset with 87,000 images of 29 classes. Empirical results indicate that with both datasets, the proposed model analysis CvT outperforms baseline models, achieving the highest accuracy of 99%, surpassing traditional CNN and transformer-based BeIT models. The findings demonstrate that CvT effectively reduces misclassifications, improves predictive confidence, and enhances generalization across training, validation, and test sets.

## Introduction

Language is the foundation of human communication, enabling individuals to express thoughts, emotions, and ideas effectively. However, for people with hearing impairments, traditional spoken language presents significant barriers, making sign language a crucial medium for communication^1^. Sign Language Recognition (SLR) has gained considerable attention in recent years to bridge the communication gap between the hearing and non-hearing communities^2^. The advancement of computer vision and AI has propelled SLR research, leading to innovative solutions that leverage deep learning for efficient and accurate recognition of sign language gestures^3^. The applications of SLR extend across various domains, including assistive technology^4^, education^5^, healthcare^6^, and human-computer interaction^7^. SLR systems can be integrated into mobile applications and smart devices to provide real-time translation of sign language into text or speech, thus improving accessibility for individuals with hearing disabilities^8^. Additionally, SLR has applications in virtual reality and robotics, where machines can interpret human gestures for seamless interaction^9^. By automating sign language translation, this foster inclusiveness and reduces communication barriers in professional and social environments^10^. The significance of such technology cannot be overstated, as it has the potential to empower millions of people worldwide by providing them with improved access to essential services and communication channels such as social media^11^.

Despite the promising advancements in deep learning, SLR still poses several challenges. Variations in hand shape, motion, lighting conditions, and background noise can significantly impact the performance of SLR models^12^. Traditional approaches have been widely used for image-based recognition tasks, but they often struggle to generalize across different gestures^13,14^. Recent developments in ViTs have introduced powerful alternatives that can better capture spatial and contextual dependencies in image and videos data^15^. However, selecting the optimal model architecture and fine-tuning it for SLR remains a critical research problem.

In this study, a combination of state-of-the-art deep learning models employed to address the challenges. The primary objective is to evaluate the effectiveness of these models in accurately classifying sign language digits. Among these models, CvT achieved the highest accuracy, demonstrating its superior performance in recognizing sign gestures. The two datasets used for this study are publicly available on Kaggle and consist of 1,712 images of hand gestures representing digits from 0 to 9 and the other is alphabetic along with space, delete and none symbols with total of 29 classes. Each class contains images of hands displaying the corresponding sign for the given image. This data set serves as a benchmark for training and evaluating the performance of proposed models. The experimental results of this research provide valuable insights into the application of transformers for SLR. The high performance obtained with CvT highlights the potential of hybrid models that combine the strengths of CNNs and transformers for improved feature extraction and classification. Furthermore, this study contributes to the growing body of research in deep learning-based visual communication, offering practical solutions for real-world implementation. By optimizing transformer architectures for SLR, aim to pave the way for future advancements in assistive technologies. The research contributions of this study shown in Table 1.

**Table 1:** Key contributions of the proposed study, summarizing the major methodological developments, experimental setups, and practical impacts introduced in this research.

No.	Contribution Focus	Contribution Description
1	Transformer-Based Modeling	Implemented and evaluated state-of-the-art transformer architectures for sign-language digit recognition, demonstrating strong suitability of vision transformers for visual-gesture understanding.
2	Dataset Experiments	Conducted experiments on two benchmark datasets: a digit dataset (1,712 images) and a 29-class alphabet–symbol dataset to comprehensively analyze model performance across gesture complexities.
3	Proposed CvT Framework	Developed a CvT-based recognition framework achieving 99% accuracy, outperforming CNNs and other transformer-based models in SLR.
4	Model Interpretability	Applied Grad-CAM to visualize class-discriminative regions, enhancing interpretability and transparency of the gesture recognition process.
5	Assistive Technology Impact	Demonstrated the potential of the proposed system to support real-time, accessible sign-language recognition for individuals with hearing impairments.

**Fig. 1:**
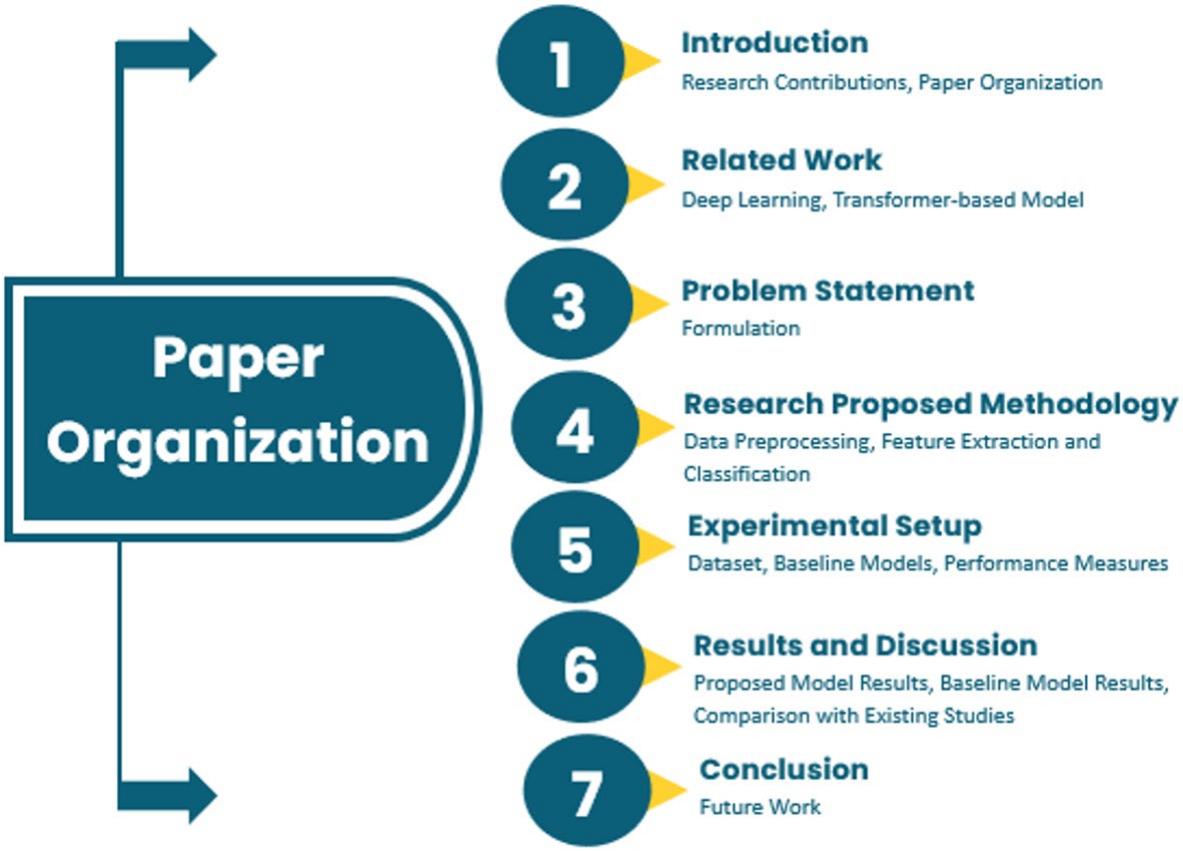
Schematic representation outlining the structural flow and section-wise arrangement of the study.

The rest of this paper, illustrated in Fig. 1, is structured as follows: Section “Related work” presents a comprehensive review of related work in SLR, focusing on deep learning and vision transformer approaches. Section “Problem statement” shares the problem statements. Section “Research methodology” provides in depth analysis of preprocessing steps used in this study, followed by Section “Experimental setup”, which describes the employed experimental setup based on dataset description along with deep learning models hyperparameters and their configurations. In Section “Results and discussion”, discuss the experimental results, comparing the performance of models. Section “Conclusion” concludes the study with key findings, limitations, and future research directions.

## Related work

Deep learning-based approaches have significantly advanced SLR by improving performance and robustness in recognizing hand gestures. Efficiency of SLR systems has been enhanced with the help of various deep learning models.

### Deep learning

In their work, Kothadiya et al.^16^ presented SLR using LSTM and GRU architecture. They showed that their method outperforms conventional models especially in terms of the sequential gesture variations. The IISL2020 dataset was used for training the model with high recognition performance. Rastgoo et al.^17^ also conducted a thorough survey over the use of deep learning models in SLR. In their work, they showed how model transition from the use of conventional machine learning models to deep learning techniques was made possible and relied on large scale datasets to improve the performance of recognition. MediaPipe’s open-source framework with deep learning-based machine learning algorithm integrated^18^. Another method dealt with efficiency at computational as well as high precision. In deep learning, robustness has been demonstrated by the implementation of hybrid models, which incorporate CNNs and RNNs, as presented in the works of Adaloglou et al.^19^ for robust recognition in SLR.

Finally, Wadhawan et al.^20^ presented work with static sign recognition using CNNs. The approach of their study includes the application of a robust model for static hand gesture classification. Sabeenian et al.^21^ also suggested a CNN based method to recognize sign using computer vision techniques. Real time recognition tasks were shown by their model to be very strong. Tolentino et al.^22^ studied isolated and continuous SLR and investigated effectiveness with different gesture recognition scenarios. Sign language videos were fed into the Inception CNN model to extract spatial features that improved recognition rate considerably in the work of Bantupalli et al.^23^. It was shown in^24^ that an SLR system with Leap Motion Controllers and machine learning can be introduced. The contribution goes beyond the physical act of gesture by integrating depth sensing tech. In Pigou et al.^25^ the CNN-based approach was also proposed by researchers on automatic sign recognition. The reviewed studies as display in Table 2, shows the progress of deep learning based SLR systems.

### Vision transformer-based SLR

Recently, transformers have drawn widespread attention for their better capacity to capture spatial and temporal dependencies for gesture-based recognition tasks compared to CNN and Transformer based encoder-decoders^26^, and so have been applied in SLR. ViTs have already been explored in several papers and compared to traditional deep learning cards such as CNNs and RNNs, as comprehensive analysis of studies display in Table 3.

**Table 2:** Summary analysis using deep learning based existing studies SLR.

Sr. No	Ref	Models Used	Dataset	Results	Limitations
1	^23^	Leap Motion + ML	ASL	85	Limited to Leap Motion-based inputs
2	^22^	Inception CNN	Video Stream	87	Requires extensive computational power
3	^21^	Deep Learning	Static Signs	86	Limited to static signs
4	^20^	CNN + Computer Vision	Custom Dataset	89	Lacks generalization to different sign languages
5	^19^	CNN	ISL	89	Not effective for dynamic signs
6	^18^	DNN	Multiple Datasets	92	High computational cost
7	^17^	MediaPipe + ML	Multiple Sign	90	Limited performance in complex gestures
8	^15^	LSTM, GRU	IISL2020	93	Requires extensive labeled sequential data

Maghari et al.^27^ present a vision transformer-based model for Arabic SLR. Turning to their study that demonstrated the feasibility of transformer models in sign recognition, they were able to match or beat traditional deep learning in terms of performance. Kothadiya et al.^28^ also introduced SIGNFORMER, a deep ViT specialized for SLR. Using transformer encoders to process sequential gesture data, they put together a model that greatly transforms encoders to perform better than the traditional architectures. In Arabic SLR, ViT and Swin transformers were investigated by Alharthi et al.^29^. To reduce model efficiency and boost the performance in real world applications, their study resorted to use of transfer learning techniques. In addition, Shin et al.^30^ used hybrid model that combines CNNs and vision transformers for Korean SLR. Work was carried out to take CNN based feature extraction target in the convolution layer. Liu et al.^31^ applied a detection transformer (DETR) with a feature pyramid network for SLR. By showing that transformers are well suited to represent usages and hierarchical feature representations. Chaudhary et al.^32^ presented a bidirectional transformer-based model for sign language translation, referred to as Signed-II. The focus of their research was the use of sign-to-text translation performance that can be improved by cross modal learning. EMPATH is an ensemble learning method leveraging MediaPipe for Bangla word level sign recognition by utilizing transformers with attention, which was introduced by Hasan et al.^33^. Using multi-modal learning, hands and facial features are combined for effective recognition. Contrary to CNN based approaches, the model outperformed on Bangla Sign Language datasets. Du et al.^34^ deployed a full transformer network with the masking future techniques and proved that transformers can handle the world-level SLR task. An end-to-end transformer-based model that jointly learned SLR and translation was proposed by Camgoz et al.^35^ called Sign Language Transformers. It was a significant leap forward in integrating these two areas into one unified system. In Cui et al.^36^, Spatial–Temporal Transformer Network (STTN) was presented for continuous SLR (CSLR). The main purpose of this model was to address low density video sequences while they are aligned with high density texts to improve the extraction of spatial temporal features^37^. The performance of STTN was compared to state-of-the-art CSLR techniques and outperforms at 95.2% accuracy on CSL and PHOENIX- 2014 datasets. But for long video sequences the complexity increased. In^38^, Sarah et al. proposed to detect sign language using multi-heading transformer attention to sign feature embedding based on SWIN model.

**Table 3:** Summary analysis using transformer learning based existing studies SLR.

Sr. No	Ref	Models Used	Dataset	Results (Acc %)	Limitations
1	^33^	Sign Language Transformers	SLR Dataset	89	High computational complexity
2	^32^	Full Transformer Network	Word-Level SLR	92	Masking technique requires extensive tuning
3	^35^	Multi-Head Attention SWIN Transformer	Chinese Sign Language	97	Requires high-end hardware
4	^37^	Hybrid CNN-Transformer	RWTH-PHOENIX-Weather	94	Real-time latency issues
5	^34^	STTN (Spatial–Temporal Transformer)	CSL, PHOENIX-2014	95	High computational complexity
6	^30^	Bidirectional Signed-II transformer	Multi-lingual SLR	94	Needs extensive training data
7	^29^	DETR (Detection Transformer)	Digital Video	90	Sensitive to background noise
8	^28^	CNN + Vision Transformer	Korean SLR	94	Hybrid models require high computational power
9	^27^	ViT, Swin Transformer	Arabic SLR	95	Limited cross-language generalization
10	^31^	EMPATH (MediaPipe-Aided Transformer)	Bangla SLR	96	Occlusion and gesture variability
11	^25^	Vision Transformer	Arabic SLR	97	Lack of real-time inference optimization

ViT-GCN was designed by Musa et al.^39^ as a gesture-based sign recognition. They introduced a graph-based feature representation technique, which adopts the model to reduce the complexity of spatial structure learning on Korean Sign Language datasets. However, despite its success, additional computational cost was needed for graph-based sign representation. Aloysius et al.^40^ presented a Hybrid CNN-Transformer Network. This research showed that joint use of a CNN for spatial feature extraction with self-attention of the transformers enhances classification performance. In addition, more deep learning-based application in diverse area shows the advancement of methods utilized in literature such as mucormycosis, author introduced the use of multi-class black fungus dataset which consists of eye, mouth, and skin infection images with a pretrained ResNet-50 model 96.12% accuracy^41^. On TORGO and Russian-voice datasets, optimized TORGO-based WaveNet model achieves 0.92 precision in dysarthria detection and can be used in real time with low-resource devices^42^. In breast cancer diagnosis, a hybrid model with transfer learning and grey wolf optimization and CNN models (ResNet, Inception) reach 0.942 precision, 0.982 sensitivity, 0.965 accuracy, and 0.971 AUC^43^.

The reviewed literature shows a clear progression from early CNN- and RNN-based SLR methods toward more sophisticated hybrid and transformer-based architectures that significantly improve spatial–temporal modeling, robustness, and recognition accuracy. Deep learning approaches such as LSTM, GRU, CNN, and hybrid CNN-RNN models demonstrated the ability to handle sequential gesture variations and extract meaningful spatial features; however, they often struggled with dynamic signs, occlusions, and high intra-class variability. With the emergence of transformer models including VIT, Swin, DETR, STTN, and multi-modal transformer frameworks, studies consistently reported superior performance, better feature generalization, and enhanced capability to capture long-range dependencies across sign sequences. Despite these improvements, existing works still highlight challenges such as high computational complexity, sensitivity to background variations, and limited cross-language generalization. Overall, the literature indicates a strong shift toward transformer-driven SLR systems, emphasizing the need for models that balance accuracy, real-time inference, and robustness across diverse signing environments.

## Problem statement

SLR is a very challenging and complex task due to variations of lighting, occlusions and intra class similarities between signs. Traditional deep learning models like CNNs have difficulty capturing long range dependencies and thin grained spatial details necessary for more precise classification^44^. Despite gains in feature extraction, ViTs lack hierarchical tokenization and such procedures to process local and global pattern as gestures respectively. The objective is to develop a model $\:{f}_{\theta\:}$ that’s map an input say $\:X\subseteq\:{\mathfrak{R}}^{h\mathrm{*}w\mathrm{*}c}$ based on labeled dataset $\:D=\left\{\right({x}_{i},\:{y}_{i}){\}}_{i=1}^{N}$ on finite set as $\:Y=\left\{\mathrm{0,1},2,\dots\:,9\right\}$ to learn a function $\:f:X\to\:Y$ for a given sample and target output. In doing so, this study uses a Convolutional Vision Transformer (CvT) model, capable of combining convolutional tokenization with self-attention mechanisms, so spatial encoding and classification performance based on function $\:{f}^{\mathrm{*}}=arg{min}_{f}\mathbb{Q}\left[\mathcal{L}\left(y,\:f\left(x\right)\right)\right]$, are raised for these challenges.

## Research methodology

**Fig. 2:**
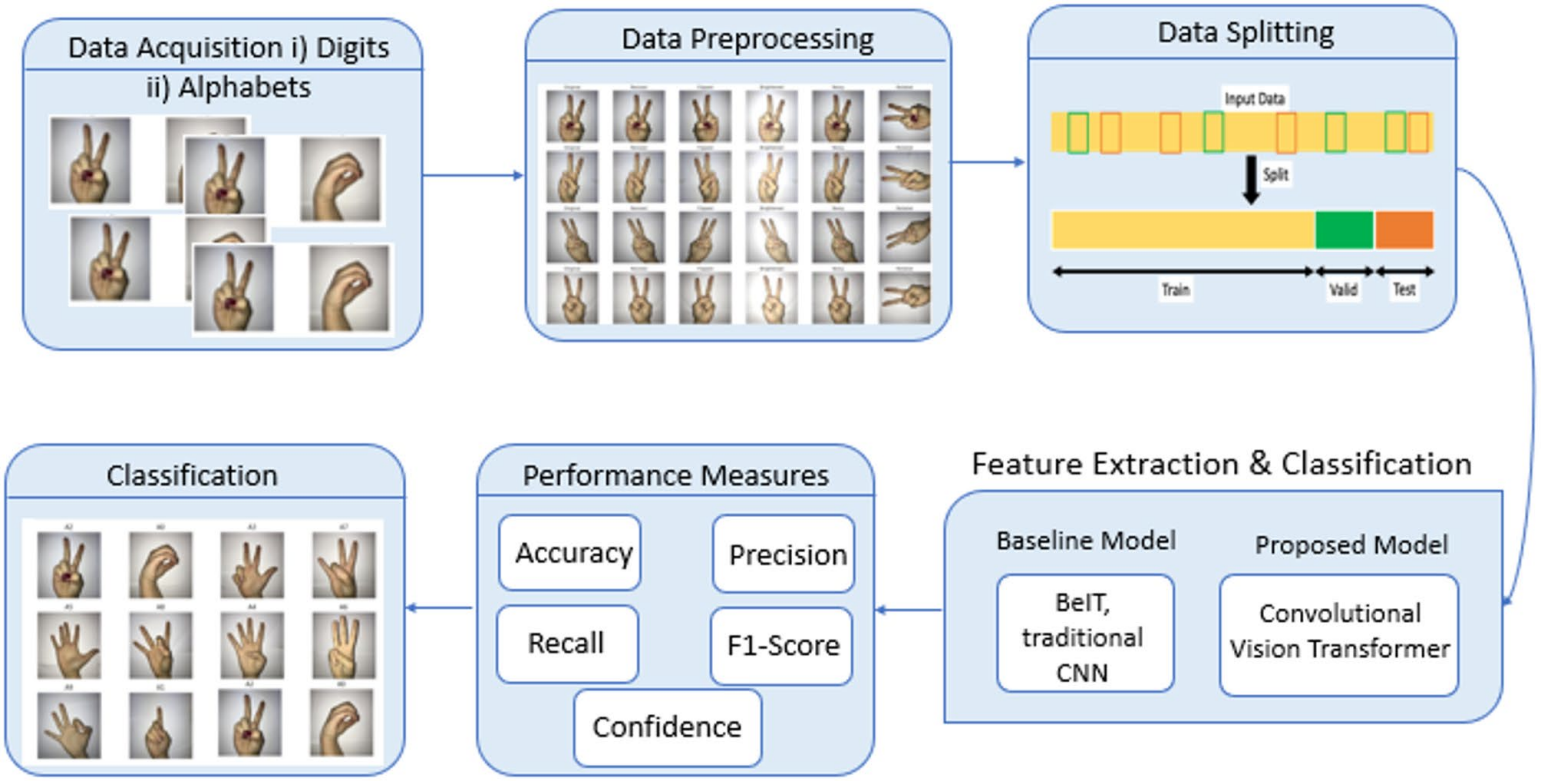
Framework showing steps followed in Proposed Research Methodology depicts the complete research workflow, highlighting each phase from data acquisition to model evaluation and interpretability analysis.

The proposed methodology for SLR is structured into three key stages, as defined in Fig. 2. The proposed model uses Hierarchical convolutional tokenization in the feature extraction phase to perform both local and global spatial feature extraction for improved sign gesture recognition with CvT^45^. Lastly, classification step utilizes transformer-based self-attention mechanism to achieve less misclassification error for gesture classification. Because of this, recognition can be performed to high performance with low computational cost rendering this an appropriate methodology for real world sign language applications.

### Data preprocessing

**Fig. 3:**
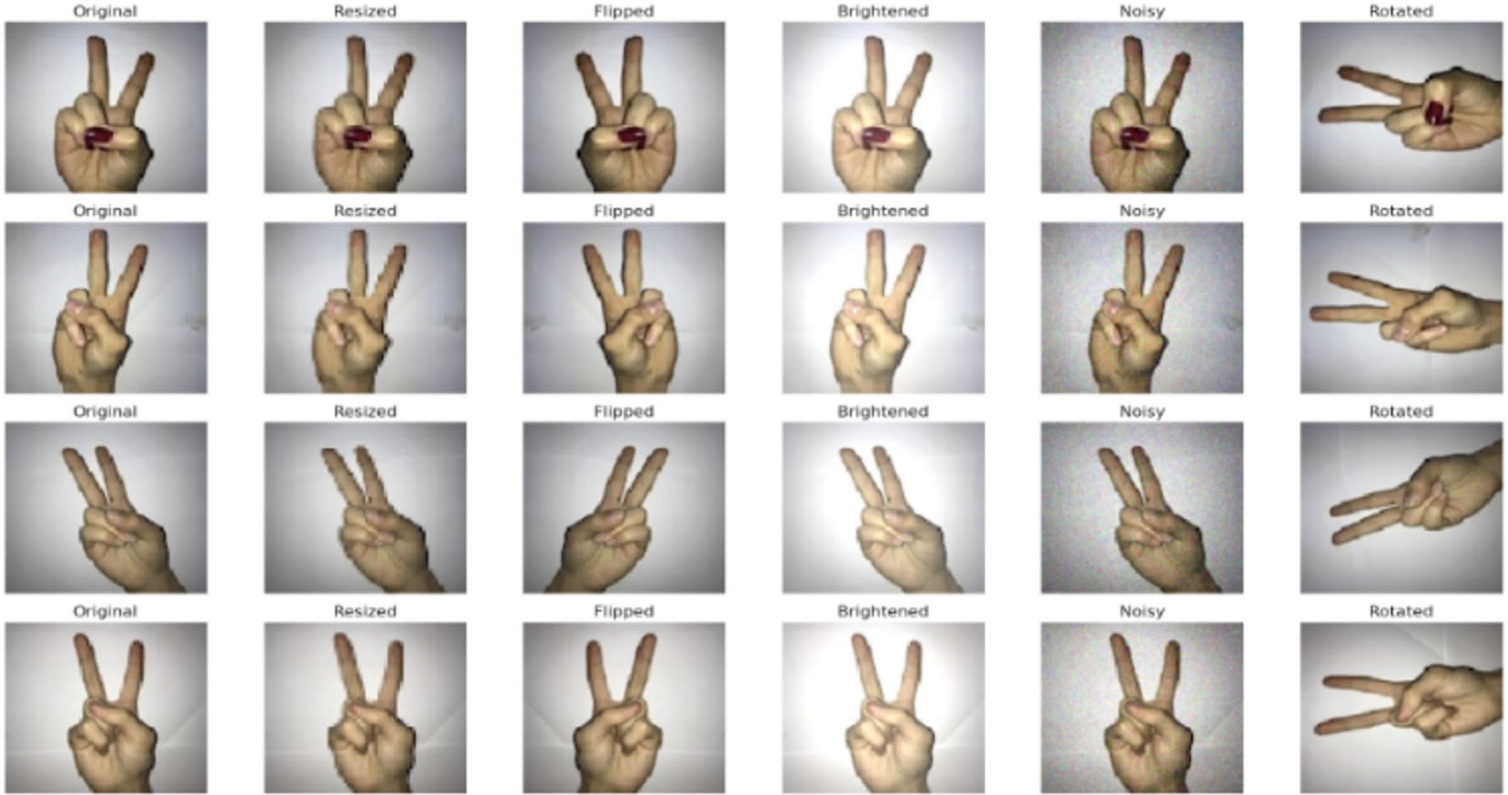
Sample images showing outcomes after applying preprocessing steps illustrating the visual quality and consistency obtained after the preprocessing phase.

Preprocessing is a fundamental step in ensuring the CvT model receives high-quality input for effective SLR. First, due to the intricate nature of the hand gestures, as well as the relative lighting conditions and even background noise, multiple preprocessing techniques were done to increase image clarity, diversity of data, and enhance the extraction of the feature^46^. Figure 3 shows the results of after preprocessing of dataset images. Transformation in the following systematic strategies has been employed.

#### Image resizing

So, for the input, it is reduced to a standard feature space by resizing each sample of the dataset such that it has a fixed resolution, maintaining a uniform feature space, as in Eq. 1^47^.

1 $$\:{x}^{{\prime\:}}=\mathcal{R}\left(x\right)\hspace{1em}\mathrm{where}\hspace{1em}{x}^{{\prime\:}}\in\:{R}^{h\times\:w\times\:c}$$

Where $\:\mathcal{R}\left(.\right)$ denotes the resizing function, and $\:c$ represents the number of color channels, $\:h\times\:w\times\:c\:$are the width, height and channel number, $\:{x}^{{\prime\:}}$ is the preprocessed input image.

#### Flipping

Random left-right flipping was applied horizontally for enhancing model generalization, computed using Eq. 2^47^.

2 $$\:{x}^{{\prime\:}}=\mathcal{F}\left(x,p\right)\hspace{1em}\mathrm{where}\hspace{1em}p\sim\:U\left(\mathrm{0,1}\right)$$

where $\:\mathcal{F}\left(.\right)$ represents the flipping operation, and $\:p$ is a randomly sampled probability from a uniform distribution $\:U\left(\mathrm{0,1}\right)$.

#### Rotation augmentation

A few small random rotations were added in a controlled range on top of the hand gestures to cover possible different angles, computed using Eq. 3^47^.

3 $$\:{x}^{{\prime\:}}=\mathcal{T}\left(x,{\uptheta\:}\right)\hspace{1em}\mathrm{where}\hspace{1em}{\uptheta\:}\sim\:U\left(-{20}^{\circ\:},{20}^{\circ\:}\right)$$

Where $\:\mathcal{T}\left(.\right)$ represents the rotation function applied with angle $\:\theta\:$ sampled from a uniform distribution.

#### Gaussian smoothing filter

Application of this to remove noise and improve clarity as well as reduce background artifacts, defined in Eq. 4.

4 $$\:{x}^{{\prime\:}}=\mathcal{G}\left(x,{\upsigma\:}\right)\hspace{1em}\mathrm{where}\hspace{1em}{\upsigma\:}=\frac{\mathrm{var}\left(x\right)}{2}$$

Where $\:\mathcal{G}\left(.\right)$ Represents the Guassian filtering and $\:{\upsigma\:}$ is dynamically adjusted based on image variance.

#### Gamma correction

To compensate for differing illumination conditions brightness enhancement was used, as in Eq. 5.

5 $$\:{x}^{{\prime\:}}={x}^{{\upgamma\:}},\hspace{1em}{\upgamma\:}\sim\:U\left(\mathrm{0.8,1.2}\right)$$

Where $\:{\upgamma\:}$ is a random brightness adjustment factor sampled from a controlled range.

To ensure that data augmentation does not compromise the semantic integrity of sign language gestures, the applied techniques were carefully controlled and designed to simulate only realistic variations encountered in practical settings. Although flipping, rotation, cropping, resizing, and image-enhancement techniques were used, each was restricted within safe bounds to preserve gesture orientation and directional meaning. Rotations were limited to small angles to mimic natural camera or head movement; while cropping and resizing were applied conservatively to maintain the spatial structure of the signing region^48^. Image-enhancement methods, including brightness and contrast adjustments, were used solely to improve robustness against lighting changes. Horizontal flipping was selectively applied only to gesture classes where directionality does not define meaning^49^. These controlled augmentations enhanced the model’s generalization ability without introducing distortions that could lead to incorrect gesture interpretation during real-time recognition^50^.

Through these preprocessing steps it is ensured that the CvT model gets high quality and diverse Learning data, that it can learn richer representations, minimize classification errors and achieve better recognition performance in real word applications^51^.

### Feature extraction and classification

**Fig. 4:**
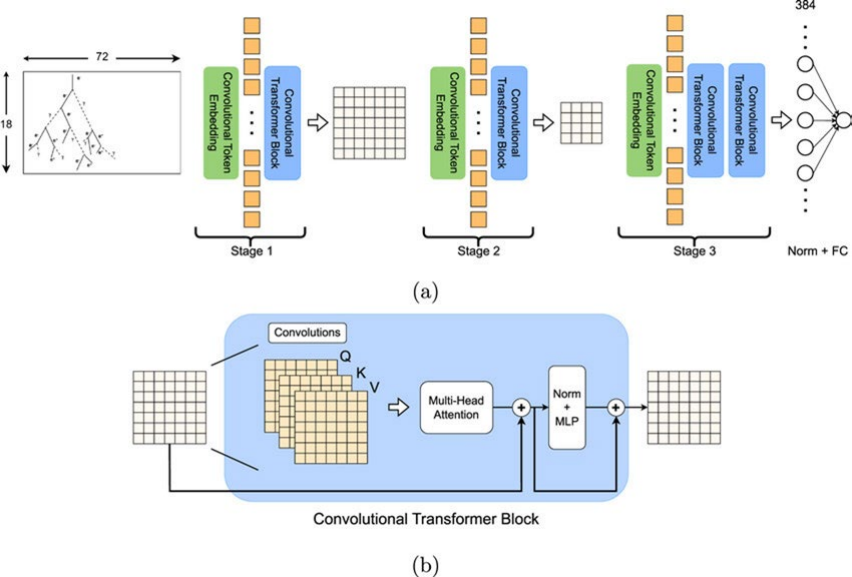
Working of proposed model CvT showing the complete working flow of the model, (a) emphasizing the hierarchical layer structure, feature extraction and fusion mechanisms, (b) and the final classification stage.

The Convolutional Vision Transformer CvT model integrates hierarchical convolutional tokenization, multi-head self-attention, and deep learning mechanisms to extract fine-grained spatial and contextual representations^52^ for SLR. Figure 4 shows the working architecture of the proposed model. Given an input $\:{\:x\in\:\mathcal{R}}^{h\mathrm{*}w\mathrm{*}c}$, CvT first applies convolutional tokenization to extract local features $\:{\:z\in\:\mathcal{R}}^{h\mathrm{*}w\mathrm{*}d}$, ensuring a structured representation, as in Eq. 6:

6 $$\:z={\mathcal{T}}_{\mathcal{c}}\left(x\right)={\sum\:}_{i=1}^{k}{\sum\:}_{j=1}^{k}{W}_{c}\left(i,j\right)\cdot\:x\left(i,j\right)+{b}_{c},\hspace{1em}\forall\:\hspace{1em}i,j\in\:\left[1,k\right]$$

Where $\:{W}_{c}\left(i,j\right)$ and $\:{b}_{c}$ are trainable convolutional filters and biases, and $\:k$ represents the kernel size. $\:x\left(i,j\right)\:is\:$Input feature value at spatial location $\:(i,j)$. Unlike traditional transformers, CvT employees hierarchal down sampling to progressively refined token representations, reducing computational overhead while retaining spatial granularity, process shown in Fig. 4 (a), computed as in Eq. 7:

7 $$\:{z}_{l+1}=\mathcal{D}\left({z}_{l}\right)=\mathrm{ReLU}\left(\frac{1}{{s}^{2}}{\sum\:}_{m=0}^{s-1}{\sum\:}_{n=0}^{s-1}{W}_{D}\left(m,n\right)\cdot\:{z}_{l}\left(m,n\right)+{b}_{D}\right),\hspace{1em}s\in\:{Z}^{+}$$

Where $\:s$ is the downsampling stride, and $\:{W}_{D}\left(m,n\right)$ represents learnable convolutional downsampling filters. $\:{z}_{l+1}$ Output token representation after hierarchical downsampling at layer $\:l+1$. This hierarchal representation allows CvT to capture both local and global dependencies in hand gestures^53^.

The multi-head self-attention mechanism follows, refining extracted features across multiple attention heads, using Eq. 8:

8 $$\:\mathrm{MSA}\left(Q,K,V\right)={\sum\:}_{h=1}^{H}{{\upalpha\:}}_{h}\cdot\:\left(\mathrm{softmax}\left(\frac{{Q}_{h}{K}_{h}^{T}}{\sqrt{{d}_{k}}}\right){V}_{h}\right)$$

Where $\:H$ is the number of attention heads, and $\:{\alpha\:}_{h}$ represents the learnable scaling factor for each head. The self-attention keys, queries and values are computed as in Eq. 9:

9 $$\:{Q}_{h}={W}_{Q}^{h}z,\hspace{1em}{K}_{h}={W}_{K}^{h}z,\hspace{1em}{V}_{h}={W}_{V}^{h}z,\hspace{1em}\forall\:h\in\:\left[1,H\right]$$

Where $\:{W}_{Q}^{h}z,\:,\:{W}_{K}^{h}z,\:{W}_{V}^{h}z\:\in\:{\mathcal{R}}^{d\mathrm{*}d}$ are head specific trainable parameters. The self-attended tokens are further through a feedforward network (FFN), as in Eq. 10:

10 $$\:\mathrm{FFN}\left(z\right)={\upsigma\:}\left({W}_{1}z+{b}_{1}\right){W}_{2}+{b}_{2},\hspace{1em}{\upsigma\:}\left(x\right)=\frac{x}{1+{e}^{-x}}$$

Where $\:{W}_{1,\:}{W}_{2}\:\in\:\:{\mathcal{R}}^{d\mathrm{*}d}$ are weight matrices, $\:{{b}_{1},b}_{2}$ are biases, and $\:\sigma\:\left(x\right)$ represents the Swish activation function.

By integrating hierarchical convolutional tokenization, self-attention refinement, and structured classification layers, CvT optimally extracts discriminative sign language features, achieving state-of-the-art accuracy in recognition task using large models^54^. CvT-Based SLR Algorithm is to efficiently classify hand gestures in sign language representing digits (0–9) using hierarchical convoluted Tokenization and transformer feature extraction. The image preprocessing, feature tokenization, multi head self-attention (MSA), and classification layers are integrated with the algorithm to enhance the model performance and generalization. By using self-attention and Convolutional layers, it allows to recognize the hand gestures accurately yet with computational efficiency^55^, working shown in Fig. 4(b). It integrates convolutional operations directly into the attention mechanism. This modification enables the model to encode local spatial context before the attention computation. In a standard transformer, token relationships are modeled globally without inherent spatial bias, whereas the CvT block introduces convolution-based token embedding, convolutional Q–K–V generation, and spatially-aware downsampling, allowing the transformer to better capture fine-grained gesture structures^56^. Additionally, the CvT block performs hierarchical feature extraction, where convolutional layers progressively reduce resolution while increasing semantic richness, something absent in regular transformers that operate on fixed, non-hierarchical token grids. Hence the flow of CvT block processes the input feature map through convolutional token embedding → convolution-based Q/K/V generation → multi-head attention → residual + norm → convolution-enhanced MLP → residual fusion to produce a spatially enriched output representation^57^.

This combination of convolution + attention provides improved locality modeling, robustness to spatial variations, and higher efficiency for gesture classification tasks.

It trains model parameters using gradient descent and loss minimization and produces optimal model predictions for the sign language images. With scalability and robustness, the structured methodology is suitable for scaling and real world-based SLR applications.

## Experimental setup

The experimental setup for this study involves deeper analysis of datasets along with evaluating the baseline models, which are further measured by standard performance evaluation metrics were used to train and test the models. In addition, the same analysis was performed on the analysis of confusion matrix and learning curves regarding classification stability, generalization ability and robustness of model on training, validation and test dataset.

### Dataset

In this study two different datasets have been utilized for experimentation.

1. Dataset 1 - SLR.

**Fig. 5:**
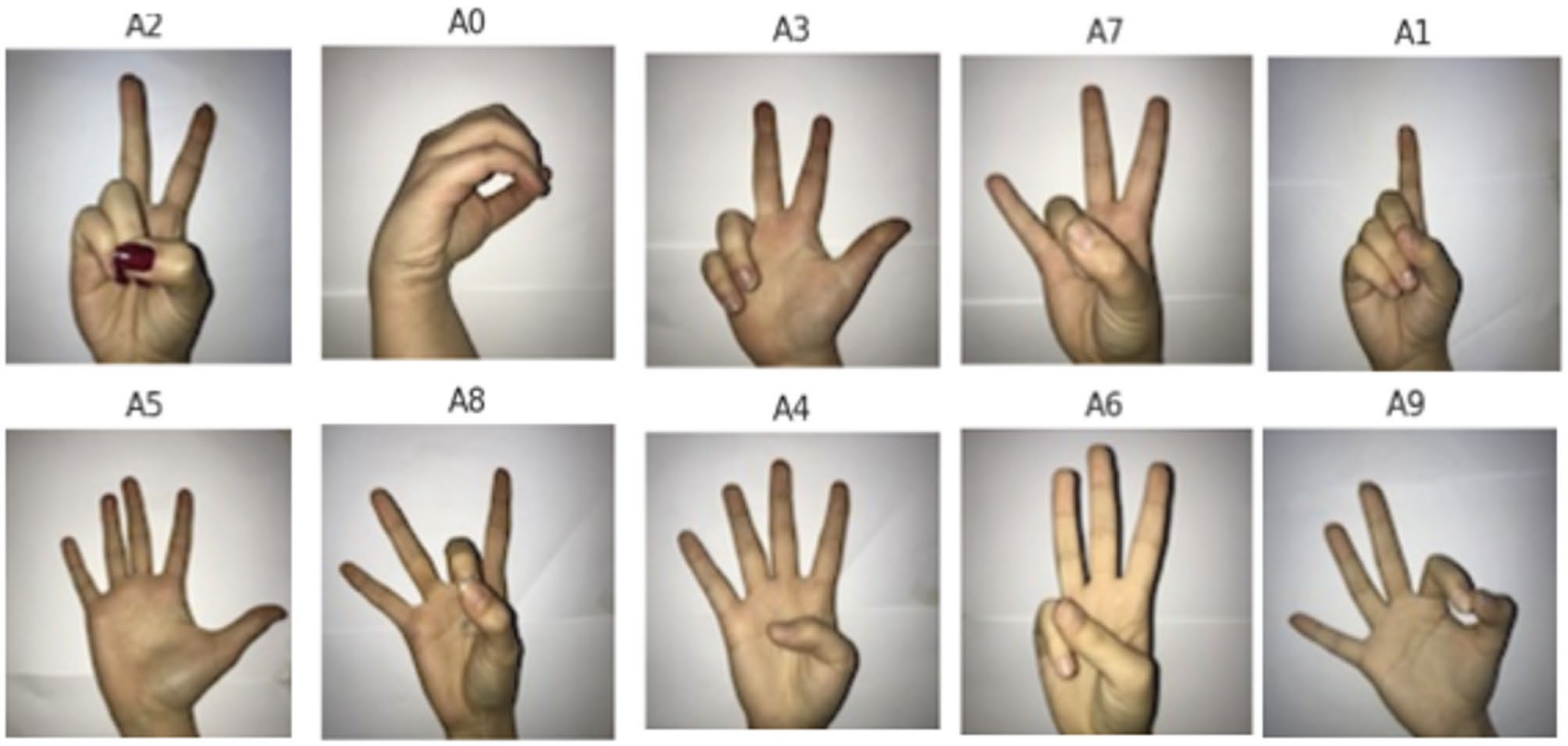
Sample Images from the dataset1 presents representative instances of each class, highlighting differences in shape, color, and texture across categories.

The Sign Language Digits Dataset, obtained from Kaggle, contains 1,712 RGB images of size 100 × 100 pixels corresponding to 0–9 for the English spoken language and 0–5 for sign language respectively. It has 171 images per class, which represent different hand positions to be digitized on the classes of the dataset, which is structured into 10, as distribution of dataset after splitting into testing and training based on ratio of 70 − 30%, display in Table 4. The dataset is based on 218 students who provided ten samples per student to cover as well as possible diverse hand variations. All the images are maintained in the same RGB color space so that the model can use the color-based feature extraction when needed. Figure 5 shows the samples images taken from dataset.

**Table 4:** Details distribution of dataset 1 images.

Classes	Actual Images	Training Images	Testing Images
A0	170	136	30
A1	171	137	30
A2	171	137	30
A3	171	137	30
A4	172	137	30
A5	172	137	30
A6	172	138	30
A7	171	137	30
A8	173	139	30
A9	169	135	30
Total	**1712**	**1370 + 342 for Validation**	**300**

2) Dataset 2 - ASL.

**Fig. 6:**
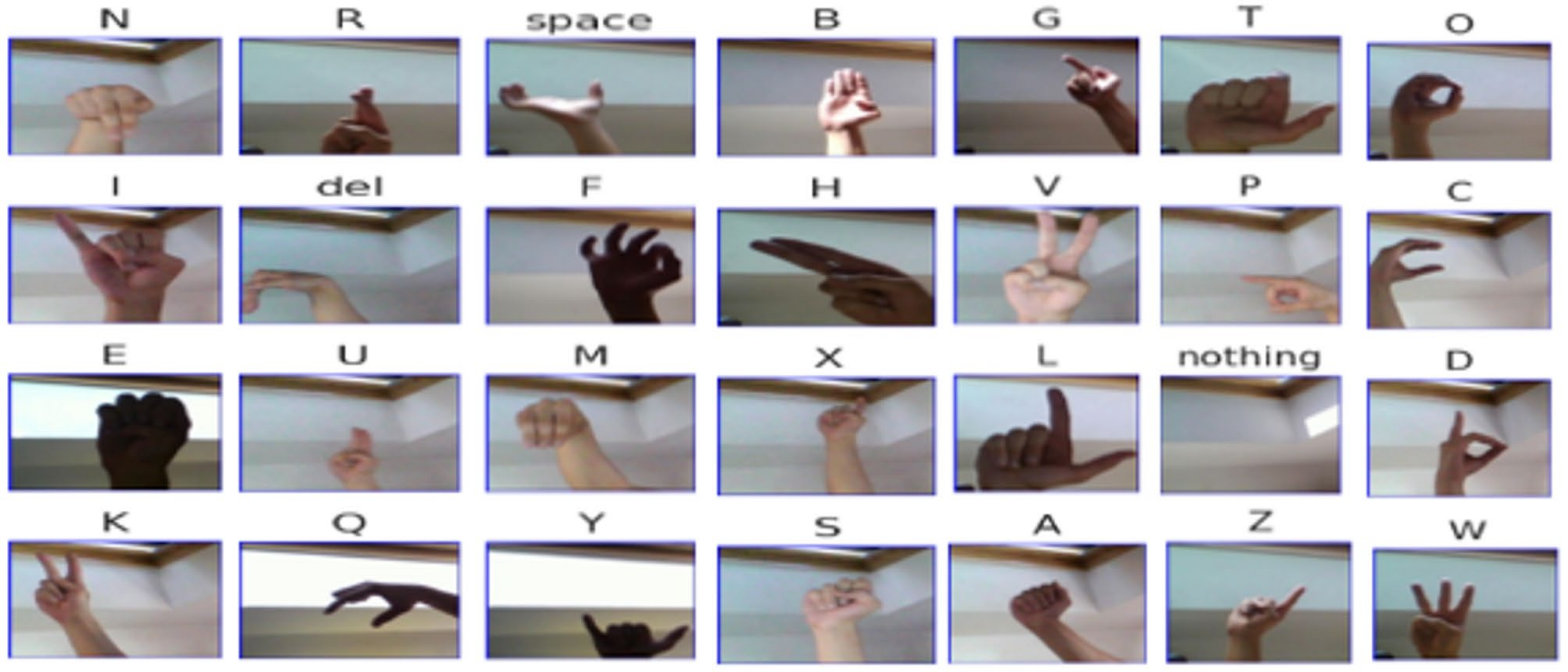
Sample Images from the dataset2 of each class to illustrate dataset structure and category-level distinctions.

Furthermore, the second dataset ASL Alphabet Image Dataset is a large visual database in which one can form images of the hand signs of the alphabet from left or right-handed individuals making it suitable for use in machine learning and computer vision research in pattern recognition and pattern analysis. There are 29 different classes in total, which include 26 English alphabet letters as well as the additional three utility classes: SPACE, DELETE, and NOTHING, as sample images from dataset shown in Fig. 6. These additional classes are especially useful for real-time and continuous gesture-spotting systems, since they allow the users to indicate pauses, corrections, and null gestures, thus improving the communicative fluency.


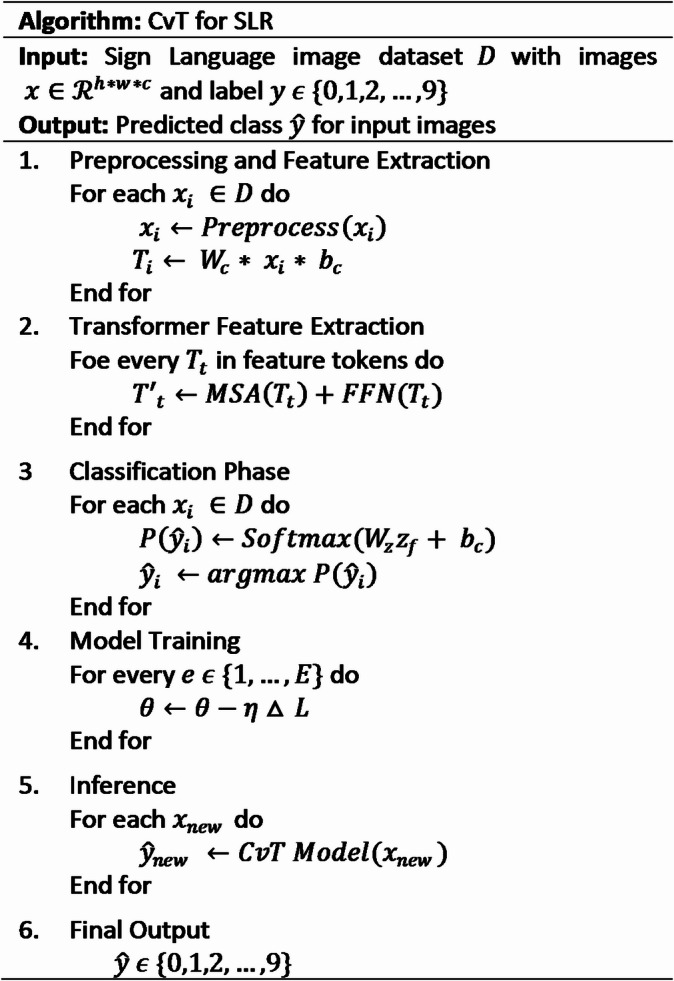


There are 87,000 (around 3,000 images per class category) training images in total. The images are normalized in 200 × 200 RGB color mode, to have the same resolution for model training, as distribution shown in Table 5. The dataset is well structured, with one directory per class, for supervised learning. Along with the large training set, a small test set has been selected that contains 29 images and evaluation against real-world or user-submitted images rather than overfitting on the curated samples. This dataset provides a rich, balanced, and structured resource for the development and evaluation of deep learning models for static hand gesture classification in ASL which facilitates the development of assistive communication systems and computer vision-based SLR systems.

### Baseline model

To provide conversions of these results on the effectiveness of the proposed MODEL, two baseline models employed and compared: BeIT and CNN.

1. Beit (bidirectional encoder from image transformers).

The Transformers based model used for self-supervised learning in the field of image processing tasks is called BeIT. In contrast to conventional vision transformers, BeIT uses the masked image modeling (MIM) scheme where image is partly masked, and the model is required to reconstruct masked parts to learn the feature representation well.

BeIT processes hand gesture images using self-attention mechanisms to understand the long-range dependencies between pixels and capture contextual relationships in the SLR. Despite that, BeIT demonstrates a more powerful global feature extraction capability compared to CvT but suffers from less efficient fine grained local pattern capturing capability due to its lack of hierarchical convolutional tokenization^58^. BeIT is then given as the computational function, defined in Eq. 11 for masked image modeling and self-attention refinement.

11 $$\:{\mathcal{L}}_{\mathrm{B}eIT}={\sum\:}_{i\in\:\mathcal{M}}|{{\Phi\:}}_{{\uptheta\:}}\left({\sum\:}_{h=1}^{H}{{\upalpha\:}}_{h}\cdot\:\left(\mathrm{softmax}\left(\frac{{Q}_{h}{K}_{h}^{T}}{\sqrt{{d}_{k}}}\right){V}_{h}\right)\right)-{x}_{i}{|}^{2}$$

Where $\:{\mathcal{L}}_{\mathrm{B}eIT}$, represents the masked image modeling loss, which measures the reconstruction error. $\:i\in\:\mathcal{M}$ refers to the set of masked patches in the input images. $\:{{\Phi\:}}_{\theta\:}\left(.\right)$ is the BeIT function that predicts missing patches based on learned representations. $\:H$ is the number of attention heads. $\:{\alpha\:}_{h}$ represent the learnable scaling factor for each attention head. $\:{Q}_{h},\:{K}_{h},\:{V}_{h}$ are the Query, key, and value matrices computed for self-attention score where; $\:{Q}_{h}={W}_{Q}^{h}z,\hspace{1em}{K}_{h}={W}_{K}^{h}z,\hspace{1em}{V}_{h}={W}_{V}^{h}z$. $\:{d}_{k}$ are the key dimensions used for skilling self-attention scores. $\:{x}_{i}$ is the ground truth corresponding to the masked patch $\:i$.

**Table 5:** Details distribution of dataset 1 images.

Classes	Actual Image	Training Samples	Testing Samples
A	3000	2100	900
B	3000	2100	900
C	3000	2100	900
D	3000	2100	900
E	3000	2100	900
F	3000	2100	900
G	3000	2100	900
H	3000	2100	900
I	3000	2100	900
J	3000	2100	900
K	3000	2100	900
L	3000	2100	900
M	3000	2100	900
N	3000	2100	900
O	3000	2100	900
P	3000	2100	900
Q	3000	2100	900
R	3000	2100	900
S	3000	2100	900
T	3000	2100	900
U	3000	2100	900
V	3000	2100	900
W	3000	2100	900
X	3000	2100	900
Y	3000	2100	900
Z	3000	2100	900
space	3000	2100	900
del	3000	2100	900
nothing	3000	2100	900
Total	**87,000**	**60**,**900**	**26**,**100**

This core computational process of BeIT refers to this equation, where the model makes use of self-attention mechanisms to reconstruct the missing patches and refining the contextual feature representations which makes it very effective in self-supervised learning in SLR.

2) Convolutional neural network.

CNNs is widely used in image classification because it can extract hierarchical spatial features from the convolutional layers, the pooling layers and fully connected layers. CNNs have been found to be efficient for edge, contour, and shape detection in SLR, and, consequently, are good for learning spatial features^59^. While CNNs do not have self-attention like the transformer base models (CvT, BeIT) they lack the capacity to capture long range dependencies, as in Eq. 12.

12 $$\:{z}_{i,j}^{\left(l\right)}={\upsigma\:}\left({\sum\:}_{m=-k}^{k}{\sum\:}_{n=-k}^{k}{W}_{m,n}^{\left(l\right)}\cdot\:{x}_{i+m,j+n}^{\left(l-1\right)}+{b}^{\left(l\right)}\right)+{\uplambda\:}{\sum\:}_{p=1}^{P}{\left(\frac{\partial\:\mathcal{L}}{\partial\:{W}_{p}^{\left(l\right)}}\right)}^{2}$$

Where $\:{z}_{i,j}^{\left(l\right)}$ represents the feature activation at position $\:i,j$ in the $\:l-th$ layer. $\:{x}_{i+m,j+n}^{\left(l-1\right)}$ denotes the input feature map from the previous layer. $\:{W}_{m,n}^{\left(l\right)}$ is the trainable convolutional filter of size $\:\left(2k+1\right)\mathrm{*}\left(2k+1\right)$. $\:{b}^{\left(l\right)}$ is the bias term for layer $\:l$. $\:{\upsigma\:}\left(.\right)$ is a non-linear activation function, such as ReLU as computed by Eq. 13.

13 $$\:{\upsigma\:}\left(x\right)=\mathrm{max}\left(0,x\right)$$

This formulates the computational function of a 2nd order CNN layer, i.e., the convolutional feature extraction and pooling, as well as the activation, as follows using Eq. 14:

14 $$\:{\uplambda\:}{\sum\:}_{p=1}^{P}{\left(\frac{\partial\:\mathcal{L}}{\partial\:{W}_{p}^{\left(l\right)}}\right)}^{2}$$

Where $\:\lambda\:$ is a regularization parameter and $\:\mathcal{L}$ is the loss function, ensuring weight optimization and controlled complexity. This encapsulates CNN’s feature extraction process, i.e., convolutions, non-linearity and weight regularization, which is important for SLR and deep learning-based gesture classification^60^.

Consequently, both CNN and BeIT serve as important baselines to compare the tradeoffs in term of deep learning harness (e.g., CNN) versus the transformer-based architecture (BeIT) in their own SLR tasks.

### Performance measures

To validate the performance of the proposed CvT model for SLR, both accuracy, Precision, Recall, and F1 score were used to assess its effectiveness, as defined in Table 6. These metrics shed light on the performance of the classification efficiency, the model robustness and the model generalization on training, validation and test datasets^61^.

## Results and discussion

Performance evaluation of the proposed Convolutional Vision Transformer (CvT) model for SLR is carried out and it out-performs conventional CNN based and transformer-based models. All of that was achieved through some very comprehensive empirical analysis to capture spatial and contextual dependencies as well as decrease misclassification error and stabilize the model’s generalization; both on training, validation, and test datasets. Further comparison with previously existing studies also shows the effectiveness of models in feature extraction, predictive confidence and classification stability. In this section, a detailed analysis of performance metrics including training convergence, analysis of confusion matrix, classification accuracy as well as confidence-based predictions to demonstrate its superiority over baseline models.

**Table 6:** Description of performance evaluation measures.

Measure	Description	Equation	Example in SLR
Accuracy	Proportion of correctly classified instances among all predictions.	$\:\frac{TP+TN}{TF+FN+FP+TP}$	If 1000 total gestures were classified and 990 were correct, accuracy = 99%.
Precision	Ratio of correctly predicted positive observations to the total predicted positives.	$\:\frac{TP}{TP+FP}$	If the model predicted 100 samples as ‘gesture 5’, and 95 were correct, precision = 95%.
Recall	Ratio of correctly predicted positive observations to all actual positives.	$\:\frac{TP}{TP+FN}$	If 100 gestures belong to ‘gesture 3’ and 97 were identified correctly, recall = 97%.
F1-Score	Harmonic mean of precision and recall, balancing both measures.	$\:\frac{2\left(Precision\mathrm{*}Recall\right)}{Precision+Recall}$	If precision is 95% and recall is 97%, F1-score balances them, giving F1 ≈ 96%.

### Results with proposed model CVT based on dataset1

The CvT model has demonstrated superior performance in SLR, achieving an overall accuracy of 99% and proving its ability to generalize across different sign classes. The results from the classification report are displayed in Table 7 with the precision, recall, and F1 score of the model for the model across all ten classes. Apart from classes such as A0, A1, A2, A3, A6, A7, and A9, all of which were perfect with precision, recall and F1 score 100%, which meant that the model correctly identified these digit votes without any mistakes.

**Table 7:** Detailed results analysis each class with proposed model.

Classes	Accuracy	Precision	Recall	F1-Score
A0	99	100	100	100
A1	99	100	100	100
A2	99	100	100	100
A3	99	100	100	100
A4	99	97	97	97
A5	99	97	100	98
A6	99	100	100	100
A7	99	100	100	100
A8	99	100	97	98
A9	99	100	100	100

**Fig. 7:**
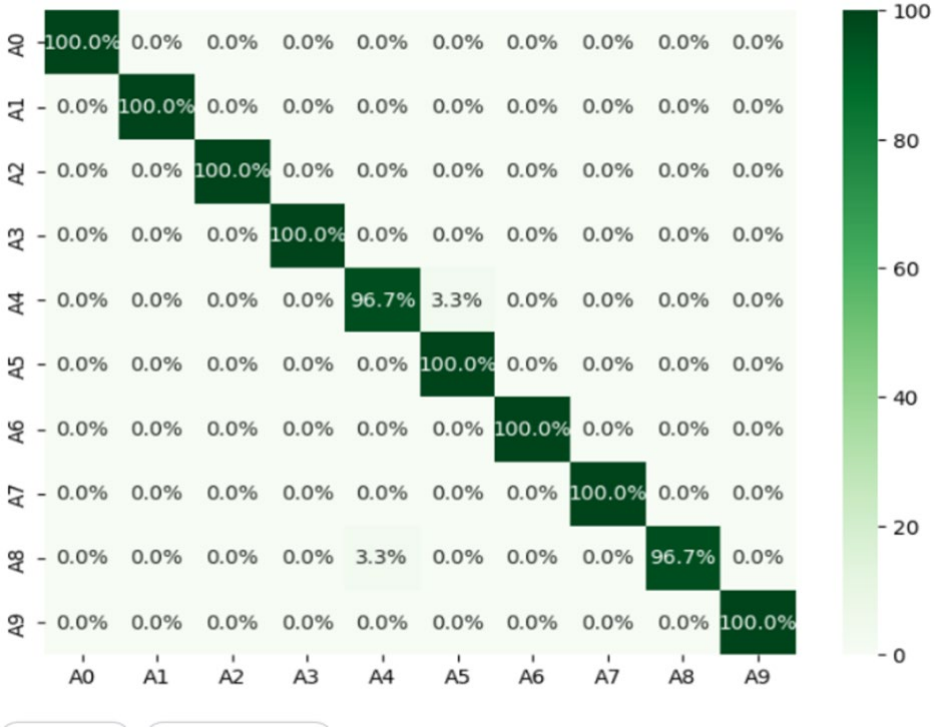
Confusion matrix Analysis of Proposed Model shows the distribution of correct and incorrect predictions for each class.

Yet A4 and A8 show slight variations in performance, and the recall comes down to 97%. This strong drop in recall demonstrates some number of misclassifications for these signs, this due to A4 and A8 sharing visual aspects of the shape, or to ambiguities in these classes that affect recognition. The confusion matrix in Fig. 7 shows that the confusion of the CvT model is tremendously high and there are only some minor confusion numbers between A4 and A8.

Probably the results of 3.3% of A4s being incorrectly classified as A8 and, conversely, 3.3% of A8s being incorrectly classified as A4s. Possible causes of such misclassification include similarity in hand positions, lighting variations, or small finger orientation variations. Such factors may result in overlapping feature spaces for these two classes, thus making it difficult for the model to classify them accurately. This however is a small issue, as the confusion matrix has a strong diagonal dominance, which indicates that almost all classes were correctly predicted by the model.

**Fig. 8:**
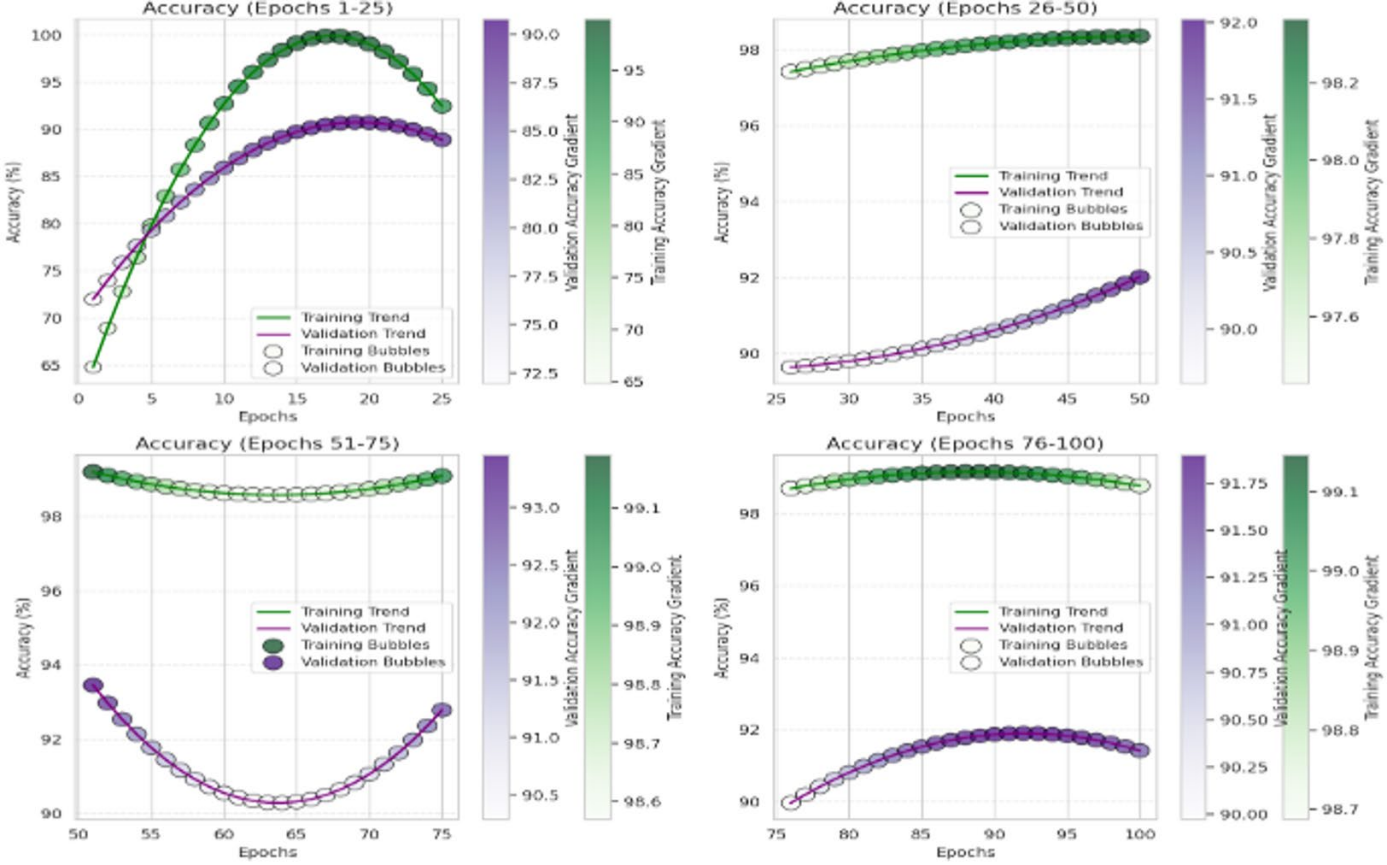
Accuracy Analysis of model over training and validation sets across epochs.

**Fig. 9:**
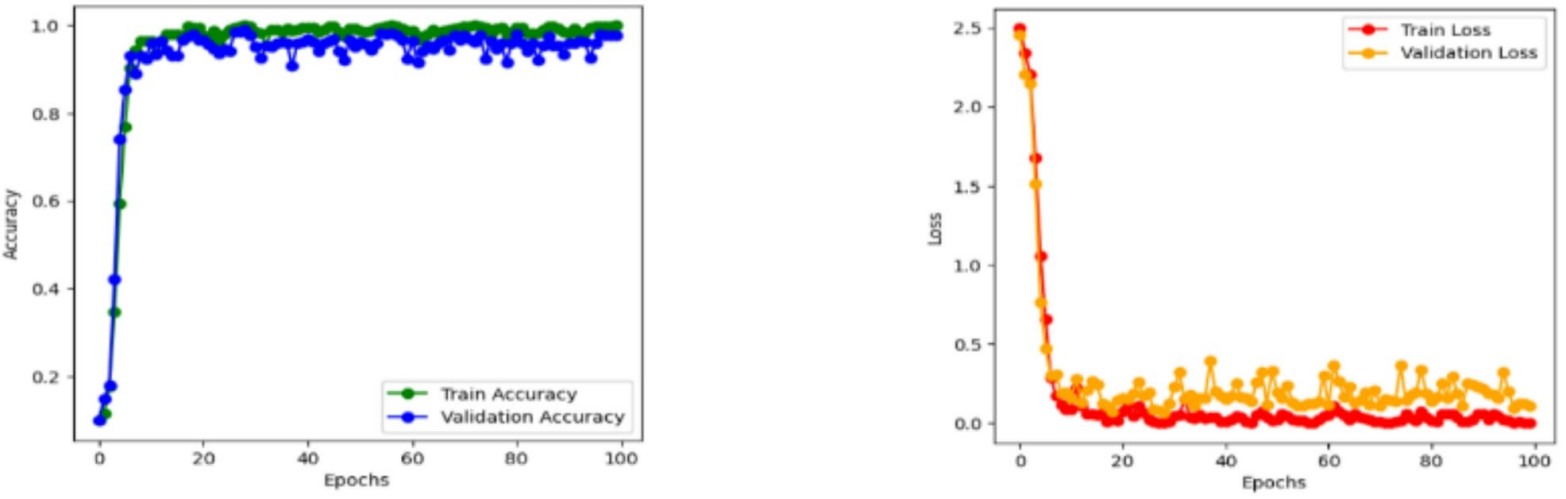
Accuracy and Loss Analysis of Proposed Model illustrates the training and across epochs, demonstrating the model’s convergence behavior and generalization performance.

Then the CvT model’s efficient learning behavior is further justified by a progressive training for multiple epochs as shown in Fig. 8. Accuracy goes up from a few percentages to over 90% in the first 25 epochs, which indicates CvT’s fast feature acquisition capability. It quickly learns essential features in sign language pattern, obviously working very effectively on its transformer based hierarchical feature extraction to grasp intricate space and temporal dependency in the dataset. The subsequent period of steady plateau of performance in the later epochs suggests that the CvT model has learned the appropriate representation for discriminating between sign gestures. The combined overall training and validation loss curves shown in Fig. 9 also give equal importance observation whereby the loss curves decreasing sharply in the start of the training and approaches zero levels by the Epoch 40. The presence of a mismatch of large deviations between validation and training loss would indicate that CvT had overfitted and therefore not exhibited strong generalization ability on seen data, which was not the case as both curves were quite similar.

**Fig. 10:**
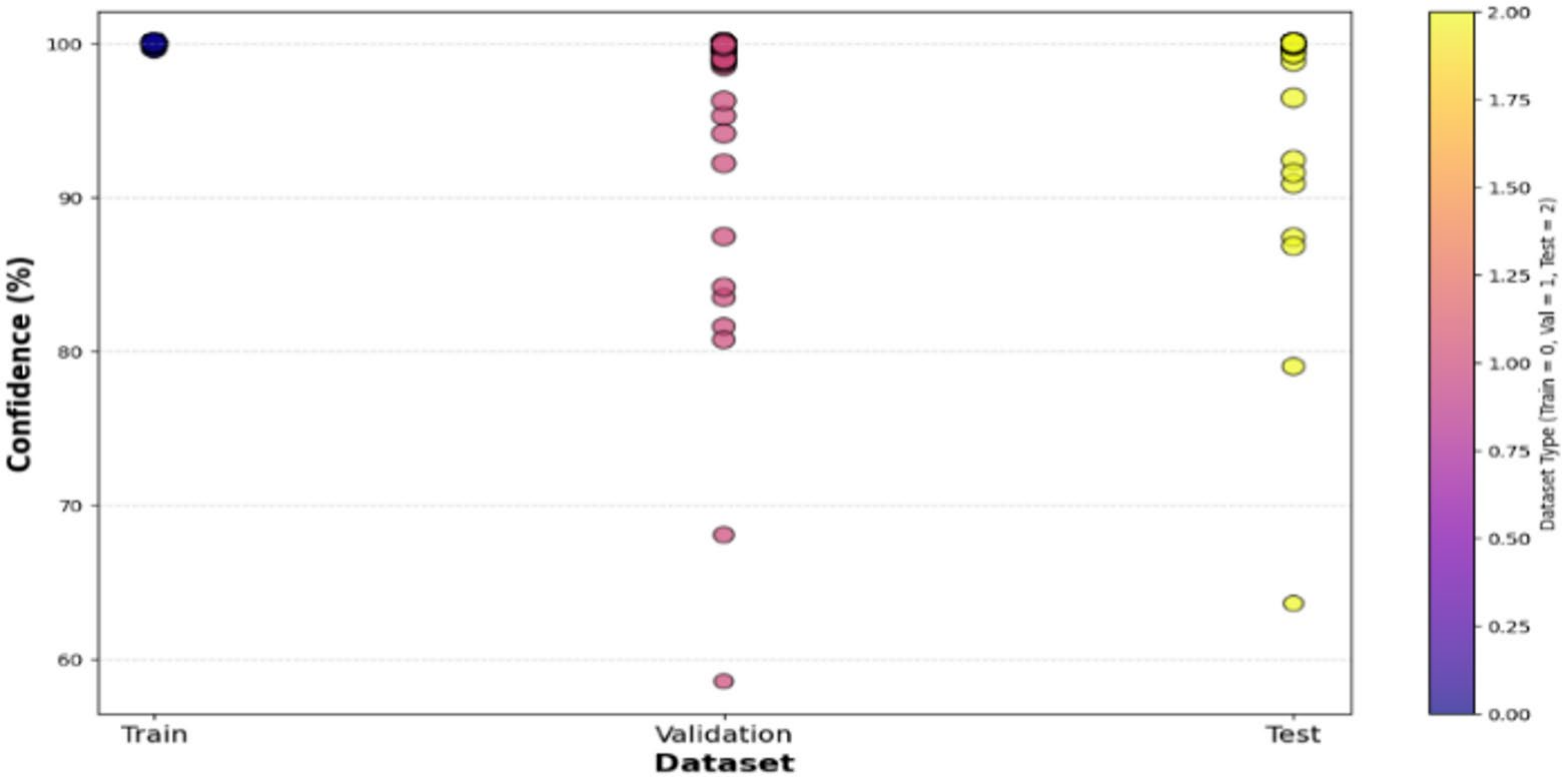
Confidence Score analysis over splitting dataset indicating calibration of proposed model.

Confidence analysis is an extremely important part of model evaluation, and it is meant to quantify the level of confidence the model has in its predictions on different datasets. The confidence distribution on training, validation, and test sets shown in Fig. 10 shows that the confidence levels are over 90% for most predictions with CvT. According to the graph, Train, Validation, and Test segments run along the x-axis, and the confidence level is shown on the y-axis. The dots on the plot represent single predictions by the CvT model, with each dot having its own vertical position and color, showing how much confidence, it has and which dataset it belongs to. The findings show that the CvT model is highly confident on the training data, as most of the confidence scores are close to 100%. The outcome reveals that the model acquired the samples and retained very minimal underfitting. Some variation can be found in the validation set, as the confidence levels are between 58% and 100%. Most validation results are confident even when the model is not fully sure, meaning the model has a strong ability to generalize. This demonstrates that the model is reliable, since most samples had high confidence in the test set and very few had confidence below 90%. With the help of gradient-based coloring, easily observe if the model is being fair and consistent when performing against different parts of the data. The good performance of the model on all splits, especially on validation and test sets, indicates it is effective and reliable for SLR. Further supporting the finding, 90% of samples have high confidence predictions, and only a few less than 80%. The select samples may feature lower confidence if their sign representations are slightly unclear or could have noise. On the other hand, this variation indicates that CvT performs very well at learning on existing samples on training data, yet there’s just a need for improvement for some seen and unseen samples in the validation and test set, with slightly lower confidence prediction. This is used to visualize if the model can perform robustly well on unseen data that are not in training data and therefore to evaluate model capability to generalize from training data. If a model displays a sudden drop in confidence across validation and test sets, then it is overfitting and such is not the case here—confidence is still quite high across all dataset splits, an indication of CvT’s strong generalization ability. However, despite this, the model is quite robust in real-world sign languages applications because it has high confidence across different datasets.

**Fig. 11:**
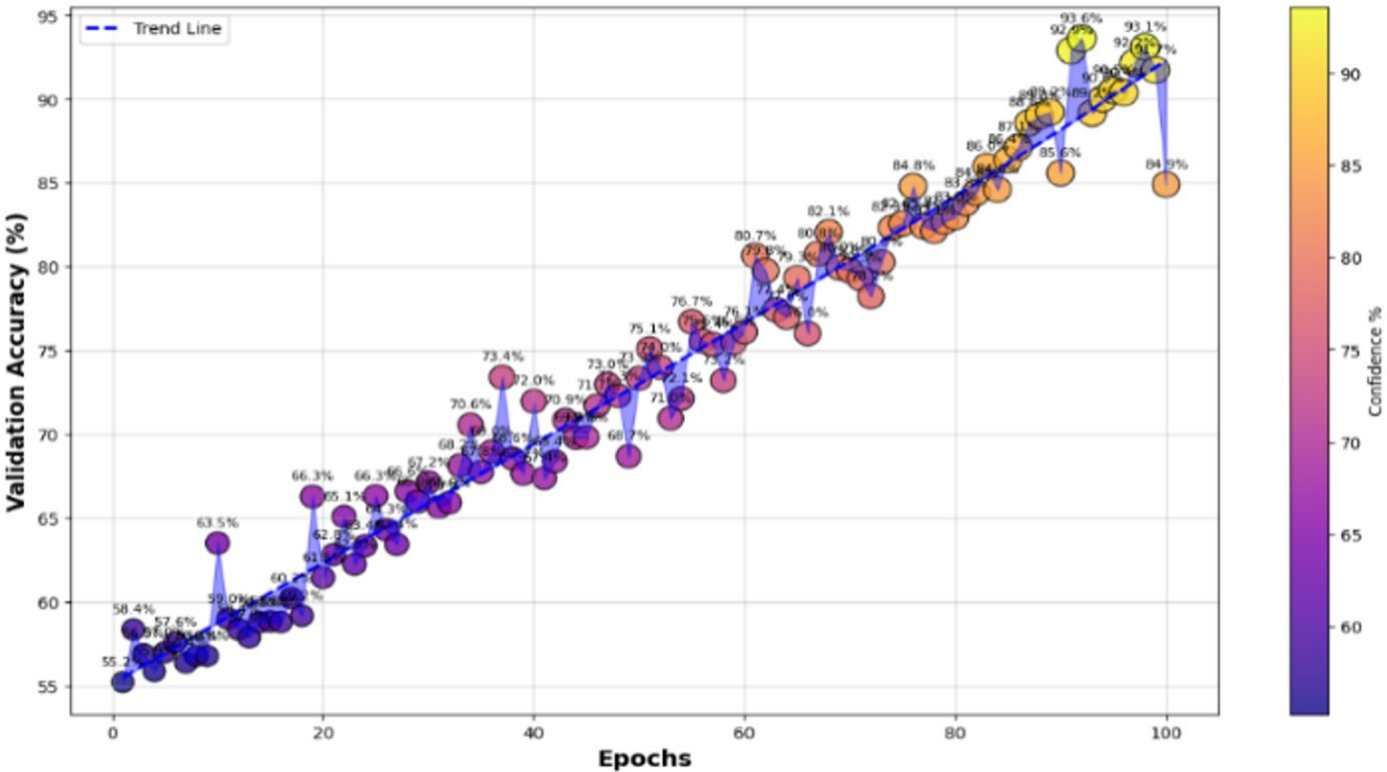
Confidence Score analysis over random samples based on epochs.

The samples-based confidence model in Fig. 11 shows the analysis a step further by giving a sense on how confident the predictions are for each sample when considered in a group of samples and provided to a one hot encoding matrix. This visualization represents the size of the bubbles as the confidence level of the individual predictions, with bubbles of bigger sizes that means more certain prediction and smaller are those that believe prediction uncertain.

The high confidence predictions across all classes for most samples due to large bubbles. Nevertheless, there are a few cases where a few smaller bubbles appear in select classes, indicating the moment where the model has lower certainty in its predictions. This plot acts to check the quality of individual sample confidence levels, which can indicate any areas which the model might need small tweaks in.

The confidence trend analysis, that analyses how certain is the CvT’s certainty over epochs, is another important observation that can be derived from it. This trend of continuous upward trend is shown by the plotted trend of prediction confidence. This consistency in the rate of confidence growth indicates that the CvT model is learning in a structured manner, and within the expected iterations the confidence grows with greater certainty in the predictions. With the training progresses, the confidence range gets smaller, and most of the predictions lie in high confidence area (95% and more), which means a well standardized and reliable model.

**Fig. 12:**
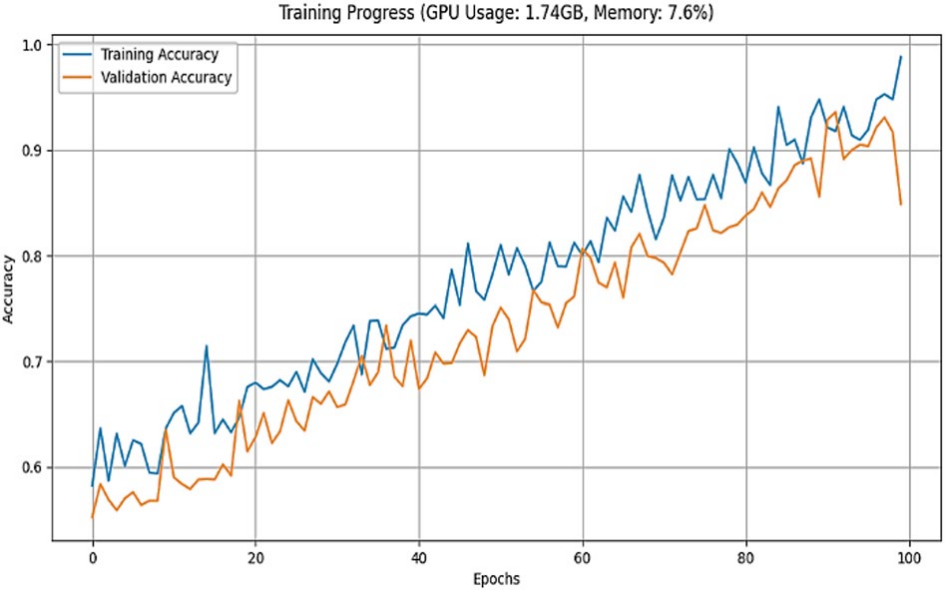
Training Time based on memory usage of proposed model showing how memory allocation impacts the overall training duration.

Confidence scores continue to trend upwards and show that CvT is making the correct progress: learning better representations, progressively sharpening its uncertainty over the evidence, and in general making progress. In addition, computational efficiency has a strong effect on the overall feasibility for deploying a model in real world applications, as shown in Fig. 12. According to the GPU utilization graph, the CvT model will utilize computational resources. This demonstrates that CvT is very efficient on both memory and processing power, and requirements of it are moderate so inferences are fast enough with small computational overhead.

**Fig. 13:**
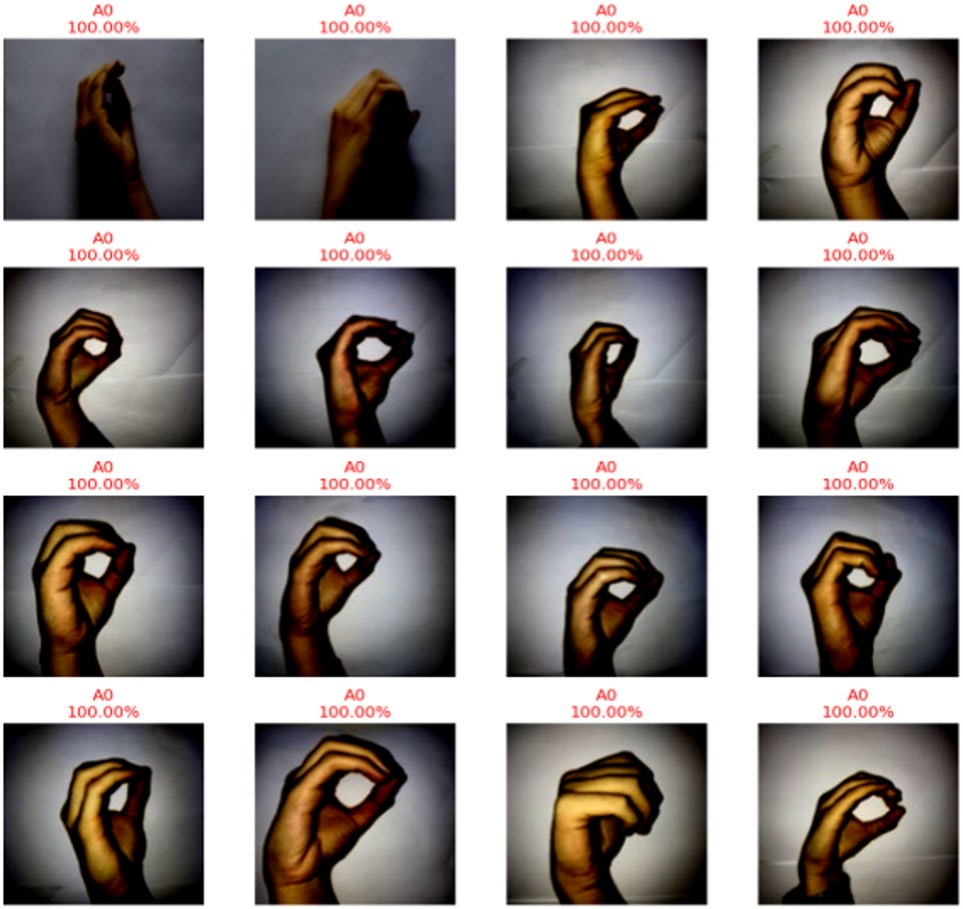
Predictive results of proposed model showing actual versus predicted class outputs along with corresponding performance metrics, illustrating the model’s classification accuracy and reliability.

As a result, CvT can strike a just right balance in between high precision as well as computation effectiveness based on hyperparameters settings as defined in Table 8, making it the great option for real time SLR software applications, such as assistive communication gadgets, embedded AI systems, and mobile applications. Overall, the CvT model has excellent prediction power, extremely high performance, good generalization across datasets and fast convergence in learning, as predictive output shown in Fig. 13. While there is a slight misclassification that A4 and A8 are in, the model showed its capability in correctly identifying the sign language digits with high confidence. Further, its excellent performance consistency as well as GPU utilization make it a very attractive implementation that is highly viable for real world deployments in assistive technology and AI communication systems.

**Table 8:** Hyperparameter setting of proposed models.

Parameter	Values
Batch Size	32
Patch Size	16 × 16
Embedding Dimension	1024
Number of Layers	24
Number of Heads	16
MLP Dimension	3072
Activation Function	GELU - Final SoftMax
Optimizer	AdamW
Epsilon	1.00E-05
Dropout Rate	0.1–0.3
Epochs	100
Learning Rate	0.00005
Weight Decay	0.01
Dropout in FC Layers	0.1
Patience	3
Split	70 − 30
Early Stopping	Patience = 10 epochs
Batch Normalization	Applied after each Conv/Attention block

### Results with baseline models based on dataset1

The CvT model evaluated with the two baseline models namely, CNN and BeIT. Specifically, these models were chosen as they have demonstrated success in image classification tasks based on images. CNN is a conventional deep learning approach with great capability in spatial feature extraction and BeIT, a transformer-based model, uses self-supervised learning and attention mechanisms. To assess the performance of these models to the same dataset and experimental setups, the same dataset is run through these models, and the results are compared in terms of performance, generalization, and computer efficiency. The results showed that CvT is superior to BeIT and CNN on the accuracy and robustness of SLR since it performed better in performance and achieved improved robustness over the signal language recognition.

1. Results with beit model.

For baseline purposes, BeIT was also applied to evaluate its capability to recognize sign language. BeIT’s performance is evaluated in terms of classification metrics and confusion matrix analysis, performance progression, loss trends and confidence-based prediction. The implications of these results are that they give more information about how well BeIT generalizes compared to CvT on sign gesture recognition. The classification report as displayed in Table 9, including results of the overall performance at 97%, suggests a strong predictive capacity in the case of BeIT. However, while BeIT is much better than CvT as a whole, it does not perform perfectly across different sign classes. Significant differences exist in the precision and recall values for certain class labels, i.e. A5 (precision = 0.83, recall = 0.90) and A4 (precision = 0.78, recall = 1.0). Some classes such as A2 and A0 are known to achieve perfect precision and recall (1.00), while others do not.

**Table 9:** Detailed results analysis each class with BeIT model.

Classes	Accuracy	Precision	Recall	F1-Score
A0	97	100	100	100
A1	97	100	93	97
A2	97	100	100	100
A3	97	100	93	97
A4	97	96	90	93
A5	97	83	100	91
A6	97	100	97	95
A7	97	100	97	98
A8	97	97	100	98
A9	97	94	97	95

This implies that BeIT can capture dependencies in spatial dimensions of sign gestures, albeit they may have greater misclassifications due to similarity of some hand postures. Although the macro and weighted average scores remain at 0.97, which is still a well-balanced model performance for all classes, the consistency is less than that of CvT.

**Fig. 14:**
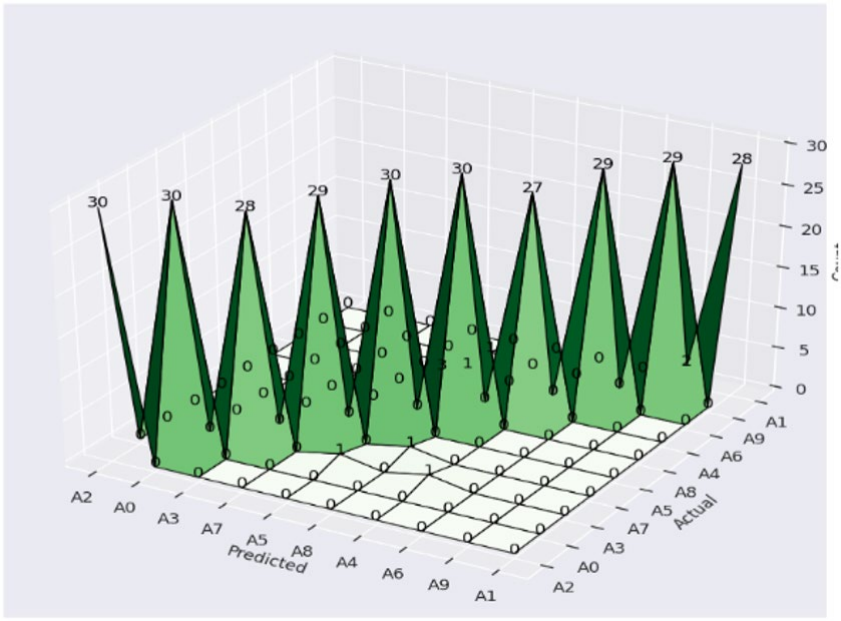
Confusion matrix Analysis of BeIT Model.

By visualizing the confusion matrix in Fig. 14, indicate the breakdown of BeIT’s predictive performance on a class level. CvT, in contrast to BeIT which shows some misclassifications, especially for A3, A7, A4, A6, A9 and A1, 3.3–10% of which were predicted to the wrong classes. It is worth mentioning that A4 has the highest misclassification rate (10%) which means BeIT cannot discern its features.

**Fig. 15:**
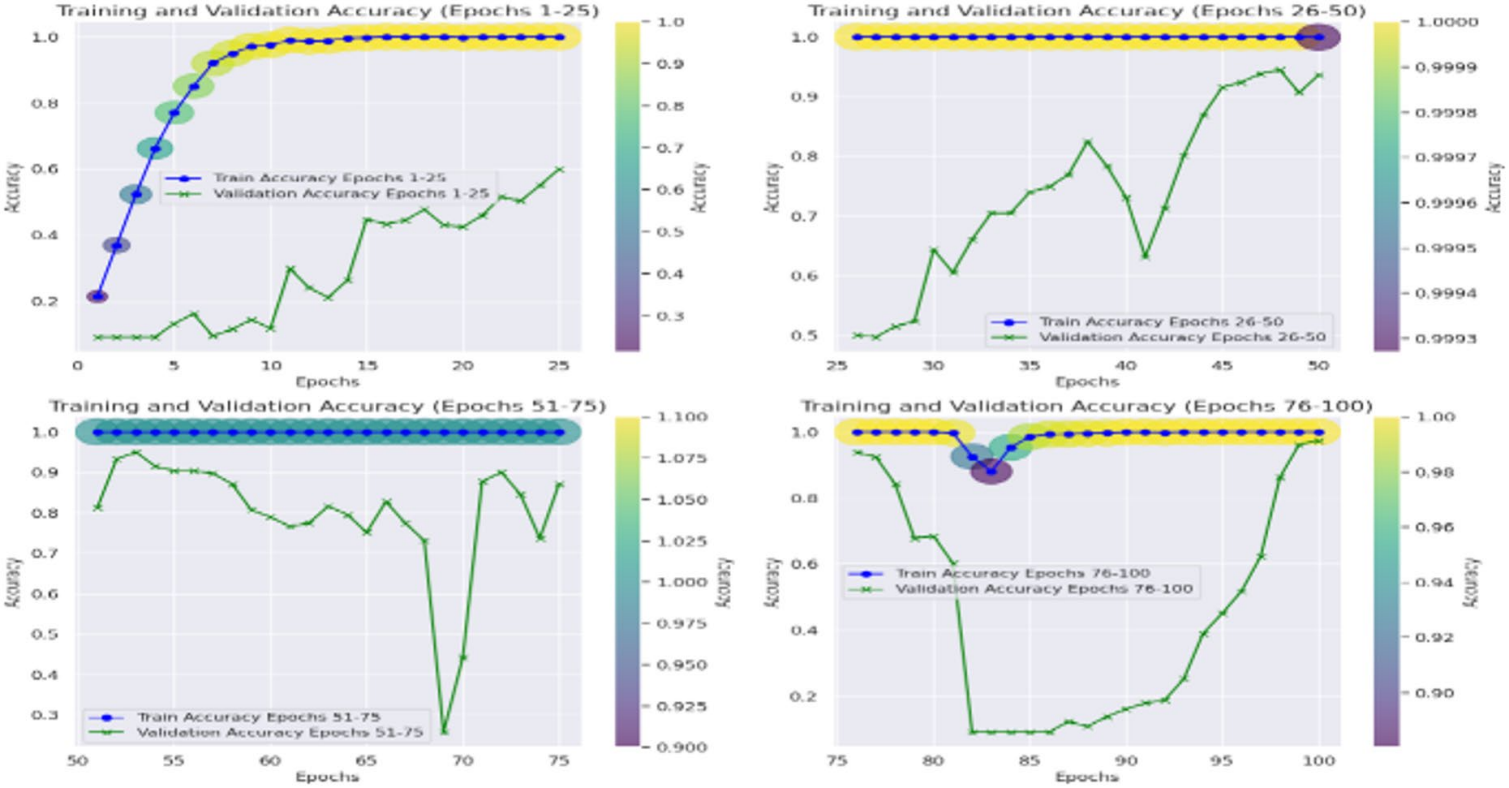
Accuracy Analysis of model over training and validation sets across epochs.

The fact that BeIT’s feature extraction ability is less strong in these cases suggests that in some cases, sign language image patches are not well taken. However, while the matrix does have these issues, the structure of the matrix is still strong diagonal, indicating that the model in general does well. A deeper understanding of BeIT’s learning curve is gained by how accurate the performance graph is over training epochs, as shown in Fig. 15. While CvT had a sharp increase in accuracy, the model started from insignificant accuracy during initial 1 to 25 epochs and increased gradually. This means that BeIT is slow to learn and has a stable end at 90% accuracy at epoch 50. Interestingly, in the later training stage (epochs 51 and beyond), the validation accuracy fluctuates greatly, which indicates that BeIT cannot maintain a stable performance in new data. Such fluctuations may signal overfitting: the model will be overly dependent on the training data and will not generalize well on samples of the validation set.

**Fig. 16:**
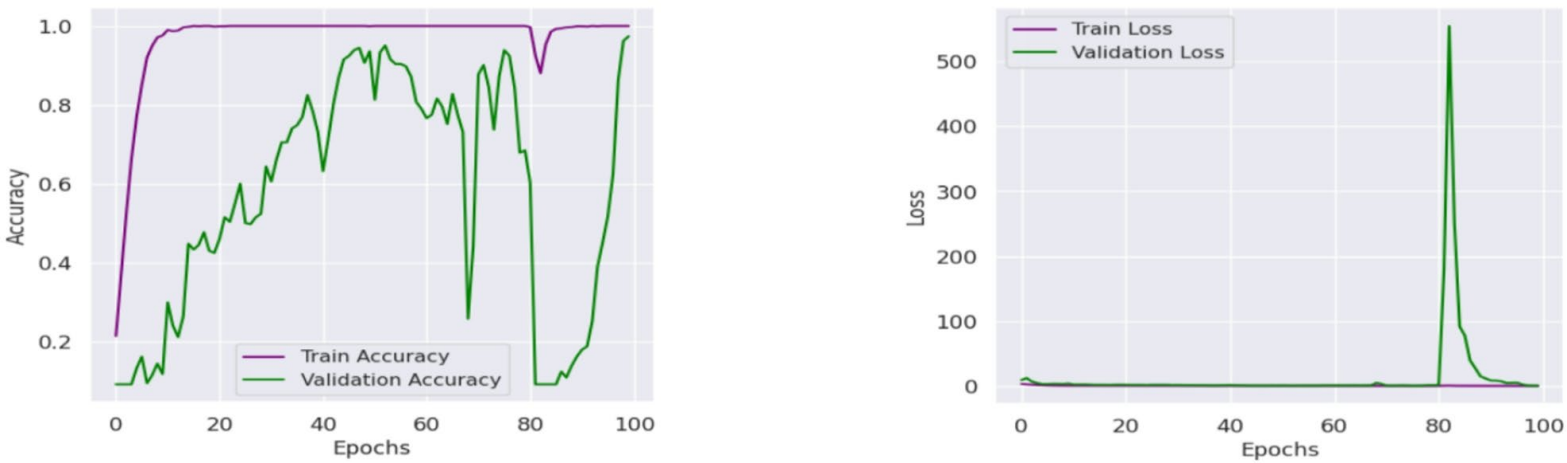
Accuracy and Loss Analysis of BeIT Model showing convergence analysis.

**Fig. 17:**
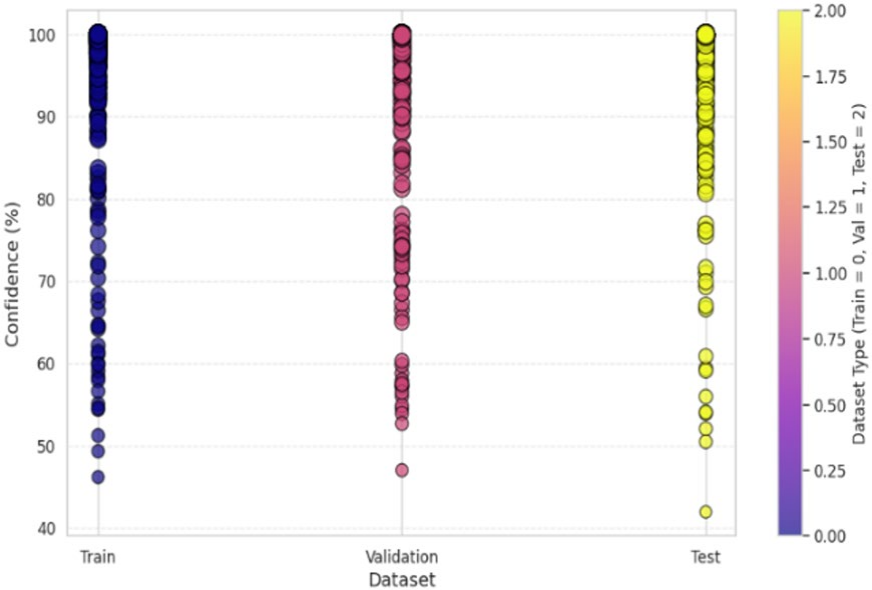
Confidence Score analysis over splitting dataset using BeIT model.

The overall resultant training and validation loss curves in Fig. 16 also support the unreliable generalization of BeIT. Upon first decreasing as expected, validation loss thereafter spikes sharply from epoch 75 onwards; this suggests that that model’s ability to generalize effectively has been lost. In fact, this is a result of overfitting, whereby the model begins to over memorize patterns in training, leaving little else. This is a major difference between BeIT and CvT, CvT sees a constant loss reduction, whereas BeIT behave more volatile in its loss. An indicator of the confidence in the predictions by the BeIT model is the confidence distribution plots. Most training samples with confidence level greater than 90% and there are more validation and test samples spread in a lower range of confidence values, some as low as 60% certainty. This agrees with the observed fluctuations of the validation accuracy and loss trends, because BeIT’s generalization is less strong than CvT’s. This shows fluctuations in validation accuracy, making this observation in alignment with the fact that BeIT proves itself to be very good at generalizing across test cases, yet it is not very robust to achieve generalization effectively on all test cases, shown in Fig. 17. Although the BeIT model has 97% accuracy, it suffers from minor instability in performance, loss, and prediction confidence in the validation and minor instability in loss and prediction confidence in the prediction.

**Fig. 18:**
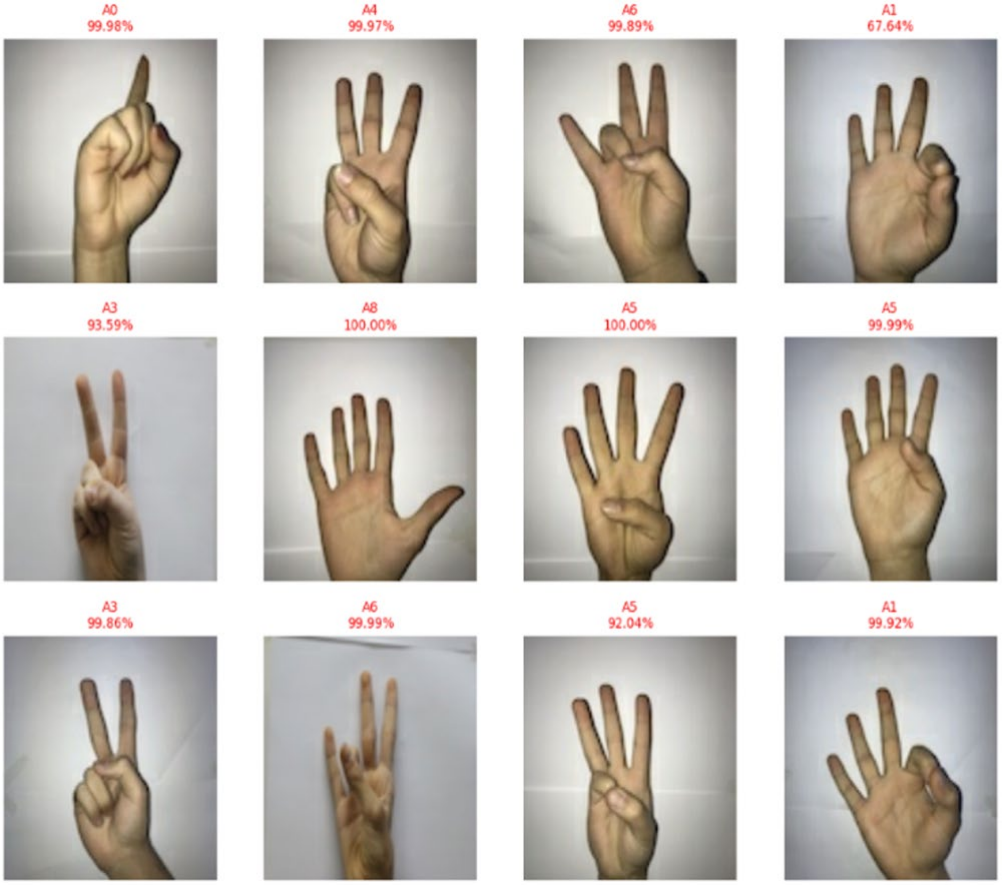
Predictive Results of Beit Model of actual vs. predicted.

In addition to the model’s inherent challenge in generalization, the confidence plotting between dataset splitting also indicates it. Almost all predictions are clustered very close to 100% certainty in training set confidence, which remains consistently high. In contrast, confidence values in the validation and test datasets have broader distribution and many samples have confidence scores between 50% and 80%. These observations suggest that BeIT is a very powerful and strong transformer-based model but lacks the robustness and stability of CvT on more complex sign gestures and unseen test cases, shown in Fig. 18.

This confirms that BeIT is very confident to train samples but becomes noticeably less sure about unseen data. Ideally, it should be a well-generalizing model, meaning that the confidence should be relatively stable (not too much) on all dataset splits; however, in this case the large confidence drop from the training to the validation/test sets indicates overfitting somewhere. It is a good memorization of training features and takes on a lot of certainty; it does not retain that much certainty when applied to new examples.

2) Results with traditional CNN model.

The baseline for SLR through CNN model was evaluated. Image classification tasks have been widely done using CNNs because they can extract spatial features effectively. In general, however, the CNN model has not performed well when comparing to transformer-based architectures such as CvT and BeIT, mainly in terms of generalization stability and predictive confidence. The classification report shows that CNN achieves an overall accuracy of 90%, which is rather high but significantly lower in performance compared to BeIT’s 97% and CvT’s 99%, as displayed in Table 10. Although the model’s macro and weighted averages (precision 93%, recall 90%, F1-score 90%) are close to moderate classification consistency, it also points out notable performance gaps in some various classes. There are some classes such as A2, and A7 and A8 that show high recall and precision scores of near perfections, whereas some others such as A0 and A4 and A6 and A9 and A1 scores in the range of 60% − 90% recall scores. A4 proves to be the most concerning drop, where recall drops to 60%, indicating that this sign gesture is particularly hard for the model to recognize and therefore misclassified on a frequent basis.

**Table 10:** Detailed results analysis each class with CNN model.

Classes	Accuracy	Precision	Recall	F1-Score
A0	90	100	87	93
A1	90	100	80	89
A2	90	100	97	98
A3	90	100	93	97
A4	90	100	60	75
A5	90	88	97	92
A6	90	79	90	84
A7	90	97	100	98
A8	90	100	100	100
A9	90	64	100	78

**Fig. 19:**
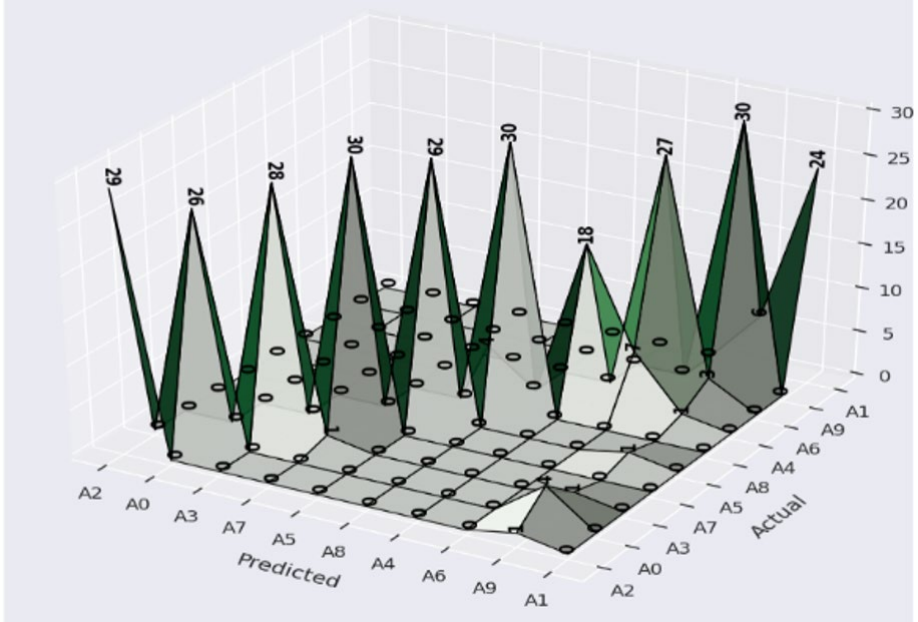
Confusion matrix misclassification Analysis of CNN Model.

Furthermore, weak recall scores are also present for A9 (64%) and A1 (80%), meaning that these signs are frequently confused with other signs. The details of misclassifications made by CNN model are also highlighted on the confusion matrix in Fig. 19. CNN’s confusion matrix in figure is more like the misclassifications in tweets that occur across many classes instead of the more well-structured diagonal dominance of CvT and BeIT. The biggest problem is A4, where there is only 60% correct prediction, and a misclassified at 23.3% samples erroneously predicted as A6. In the same way, A0 is misclassified 13.3% of the time, and A9 with A1 20% of the time. While errors are due to CNN being less capable of extracting a feature compared to vision transformers, these errors are explained. CNNs implement such hierarchies as convolutional layers, which are challenging to retrieve fine grain differences of hand gestures, while self-attention mechanisms of Transformers, such as CvT and BeIT, use self-attention to capture complex spatial relationships.

**Fig. 20:**
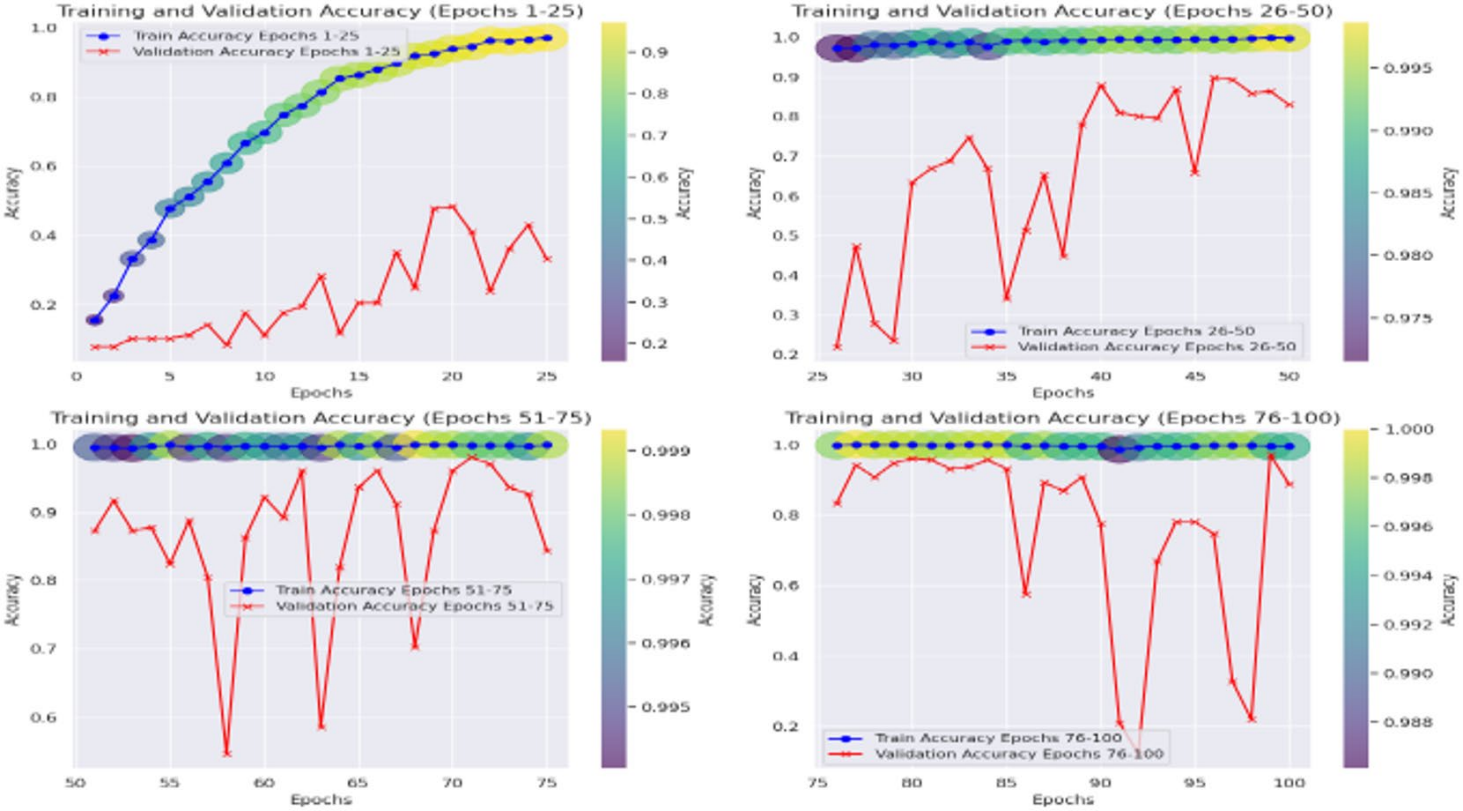
Accuracy Analysis of model over training and validation sets across epochs.

Training and validation accuracy curves across the epochs in Fig. 20 show how seriously CNN model is fluctuating during learning. CNN on the other hand is very volatile in terms of its validation accuracy across all the epochs. Accuracy increases steadily only during epochs 1–25 and validation accuracy is less reliable. For epochs 26 ~ 50, CNN obtains 90% of the accuracy but the validation checkpoint is not stable, therefore it seems that the model less generalizes consistently across unseen data.

**Fig. 21:**
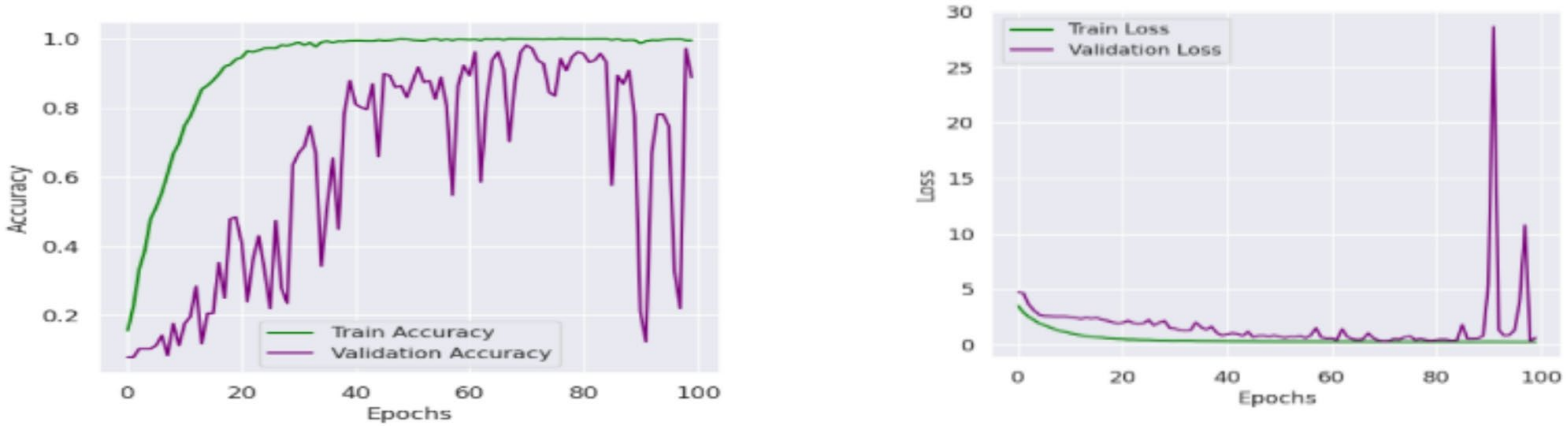
Accuracy and Loss Analysis based on training and validation of CNN Model.

**Fig. 22:**
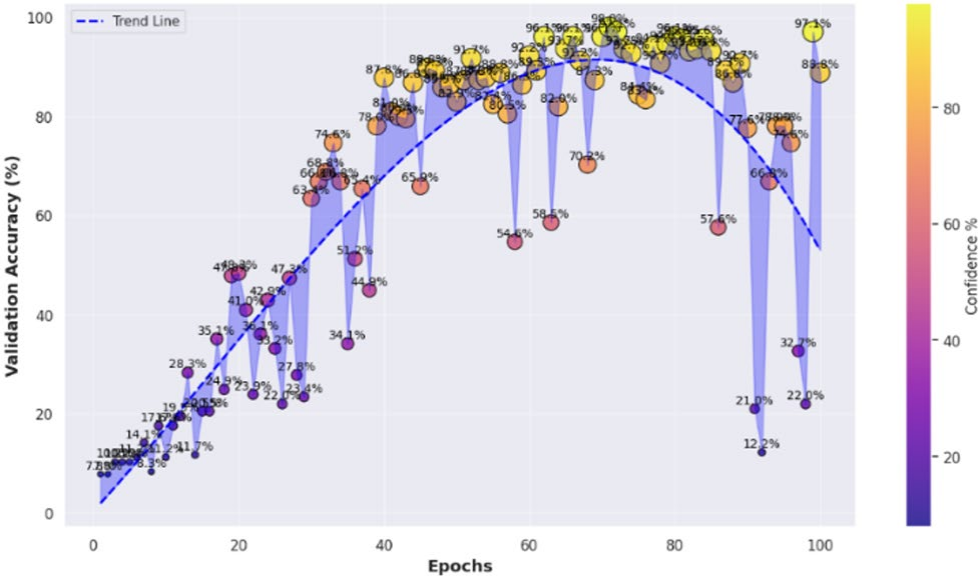
Overall Confidence Score analysis over epochs.

The accuracy plots are further backed by that of the training and validation loss curves in Fig. 21. The training loss goes down steadily, while a trend of increasing and super/very large spikes in the validation loss after epoch 80. This implies that CNN overfits on the training data; it memorizes patterns and not feature that are generalizable. Confidence prediction is another way to understand CNN’s reliability. Further confirming CNN’s instability, the accuracy confidence trend analysis confirms that the confidence scores are highly variable across epochs of training, as shown in Fig. 22. The method itself offers deep insight into the reliability and stability of the predictive of the traditional CNN model across different datasets and samples.

**Fig. 23:**
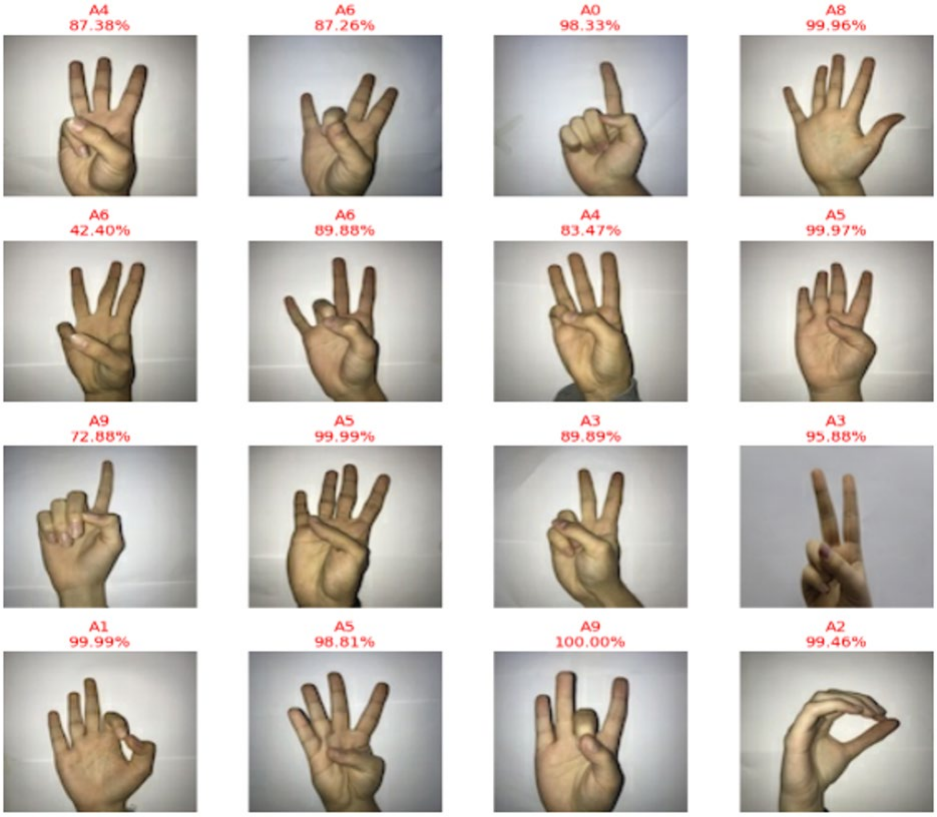
CNN model based on actual vs. predictive results.

In contrast to CvT and BeIT, which had more stable confidence levels in predictions, CNN showed very scattered confidence scores although its classifications are widely scattered. The model increases its confidence in an increasing fashion until the end of the training stages where the confidence follows erratic patterns with several dips and spikes in training. On the other hand, CvT maintains a smooth, upward and consistent trajectory of confidence, which is more reliable in models. However, traditional CNN model is not quite as efficient as the CVT and BeIT transformers that this replaces, which are known to be considerably better. The low overall accuracy (90%), misclassifications of A4, A9 and A1 (considerably) and typically unstable trends of validation accuracy, highly erratic loss values and sparsely concentrated confidence predictions indicate that CNN is unable to carry out robust feature extraction and generalization that is critical to achieving high accuracy for SLR, predictive results are shown in Fig. 23.

**Fig. 24:**
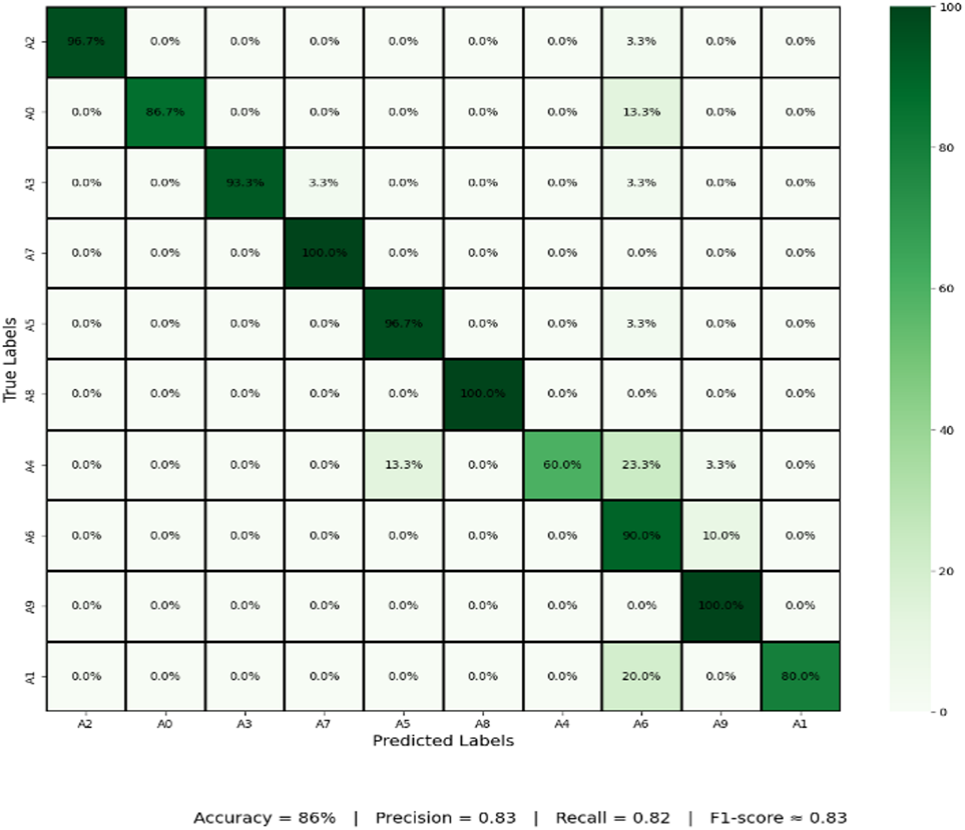
Confusion Matrix Analysis using DenseNet model.

The DenseNet model effectively recognizes sign-language gestures by analyzing dense connectivity, where each layer receives feature maps from all preceding layers, enabling efficient feature reuse and stronger gradient flow, but less efficient then CNN model shown in Fig. 24. This architecture helps the model capture subtle spatial–temporal variations in hand shapes, orientations, and joint movements essential for sign-language interpretation. The experimental results demonstrate that DenseNet achieved strong recognition performance, with an accuracy of 86%, precision of 0.83, recall of 0.82, and an F1-score of approximately 0.83, indicating balanced predictive capability and reliable classification across gesture categories.

### Comparison of the proposed model with baseline models

**Fig. 25:**
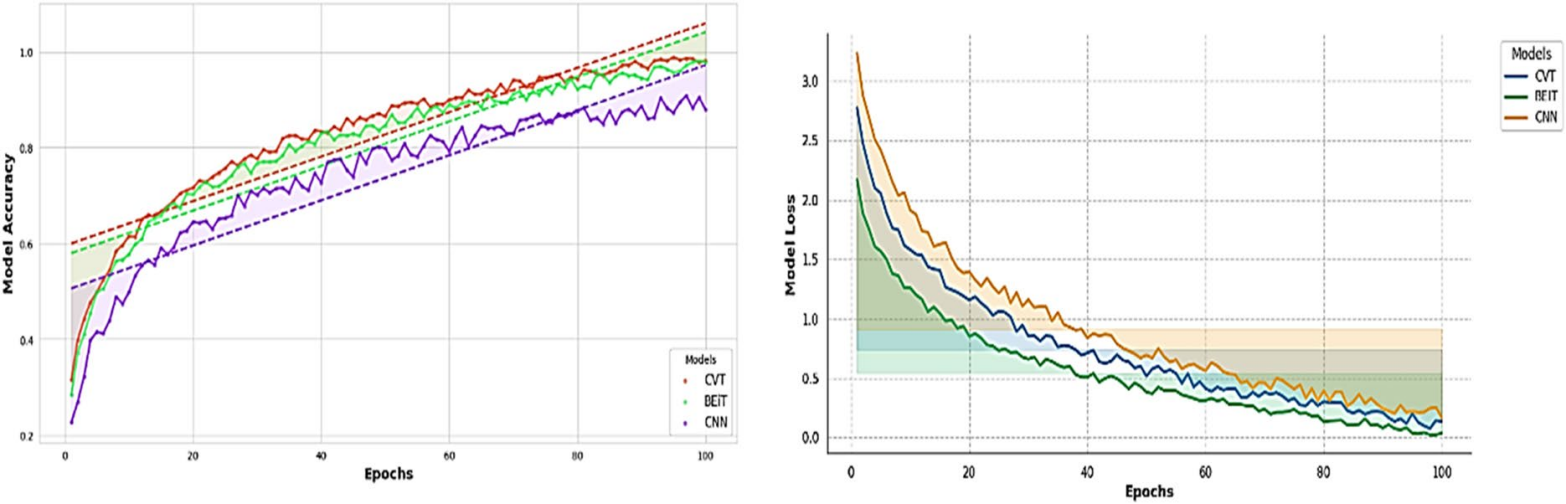
Comparative analysis of all applied models’ accuracy and loss.

Typically, CvT model has shown a significant performance margin, that is, higher accuracy, better generalization, enhanced predictive confidence, and better stability over training epochs compared to the baseline models, CNN and BeIT. CNN and BeIT act as strong benchmarks, however, the results show that the hierarchical feature extraction based on the transformer of CvT improves the learning ability in SLR. It can also be seen as one of the most striking differences in terms of accuracy and loss combined with graphs in Fig. 25. BeIT (97%) and CNN (90%) are not as good as CvT (99%), which achieves an almost perfect match, as displayed in Table 11. Classification reports show that the precision and recall of CvT are highly consistent in all the sign language digits, CvT fares a bit better than CNN and somewhat worse than BeIT for some misclassification cases. These findings are further supported by the confusion matrix visualizations which demonstrate CvT with nearly perfect diagonal dominance showing few misclassifications, BeIT with moderate confusion among a few classes and CNN with wide spread of misclassification, especially for A4, A9 and A1.

**Table 11:** Detailed results analysis with applied model.

Models	Accuracy	Precision	Recall	F1-Score
DenseNet	86	83	82	83
CNN	90	93	90	90
BeIT	97	97	97	97
CvT	99	99	99	99

CvT also proves to have more efficient and stable learning progression than accuracy. Moreover, loss curve analysis reveals that CvT has low and stable validation loss shown in figure, whereas CNN has large and sporadic spikes in the loss above epoch 80 which indicates poor generalization and overfitted tendencies. Moreover, confidence analysis breaks down CvT as the most reliable model out of the three. A confidence plot demonstrates that CvT is to remain a high confidence throughout training, validation, and test dataset, indicating that it generalizes well on unseen samples.

Compared to BeIT, which is relatively stable, it is however a broader confidence spread on certain test cases. However, CNN appears to have much lower confidence levels, with the predictions scattered excessively and the certainty in some of classes noticeably missing. A major advantage of CvT goes beyond computational efficiency and feature extraction, which is also another. CNN uses convolutional kernels to focus on spatial views, but CvT instead makes use of self-attention mechanisms to improve at determining long range dependencies better. Like transformer-based BeIT, however, its feature extraction isn’t as fine grained as CvT through modeling hierarchical convolutional embeddings.

Hence, CvT surpasses CNN and BeIT both in terms of representation learning and achieves a higher classification accuracy, better generalization as well as more loyal predictions across datasets. This study uses data augmentation which enables the proposed model to be more robust and increase its ability to generalize. The model is trained to learn invariant representations by artificially making the training samples more diverse by transformations including rotation, flipping, scaling and changing brightness, which helps it adapt to other changes in real-life situations^62^. The addition of augmentation methods also led to an observable increase in the accuracy and stability of classification and minimized the overfitting and variance between the training and testing results.

Finally, the CvT model demonstrates by a large margin that it is the most impactful method for SLR, outperforming CNN and BeIT in all the considered performance measures. Although it has a lower accuracy, the model has more stable generalization, stronger confidence distribution, and it is more efficient in feature learning, which makes it best suited for real world deployment.

### Results with proposed model CVT based on dataset2 - ASL

To explore the generalization capability of the proposed CvT-based model beyond digit recognition, further conducted a second set of experiments on the ASL Alphabet dataset, which contains 29 representative classes. The results analysis to follow gets into the details of model performance on this alphabet-based dataset, both in terms of its ability to predict the ground truth (predictive accuracy and class-wise performance); as well as its ability to generalize its prediction behavior on the alphabet signs to the larger expanded gesture vocabulary. The CvT model yielded an overall accuracy of 99% and high per-class precision and recall implying its generalization and learning capabilities. Classes like B, SPACE, DEL, X, V, W, D and J have perfect or close to perfect classification rates (around or equal to 100%), which validates that the proposed model can capture consistent and discriminative features with respect to these signs. These signs may have specific spatial or contour related properties that can be mined by the hierarchical attention layers of the CvT model.

Despite this high classification performance, a certain class showed some misclassification with presence of significant off diagonal values in the matrix. For example, the ‘I’, ‘M’, ‘O’ and ‘Z’ classes demonstrated some misclassifications. In some rare cases (about 1.1% and 0.6%), the letter ’I’ was mis corresponded with adjacent letters such as ‘L’ and ‘J’, which is possibly caused by the similar hand shape structures or occlusion. In the same way, ‘M’ and ‘Z’ classes, having more complex finger positions, exhibit noisier curve and mild confusion with classes such as ‘N’ or ‘W’, however they still recorded classification accuracy of more than 96%.

Most interestingly, even the most complex and ambiguous options (‘Q’, ‘O’, ‘Z’) retained a classification accuracy of around 92–94%, indicating the strong capability of the CvT model in modeling fine-grained features. This robustness can be explained by the fact that CvT involves convolutional tokenization and multi-head self-attention pipelined together, which enables it to capture both local spatial information and the global context information across the images. The limited error margins seen are small and isolated and in no way detract from the overarching ability of the model. The high prediction confidence and low misclassification rates of the model make it a reliable and effective approach for real-time ASL recognition. Therefore, according to the confusion matrix, CvT achieves state-of-the-art results on both accuracy and generalization across all categories.

**Fig. 26:**
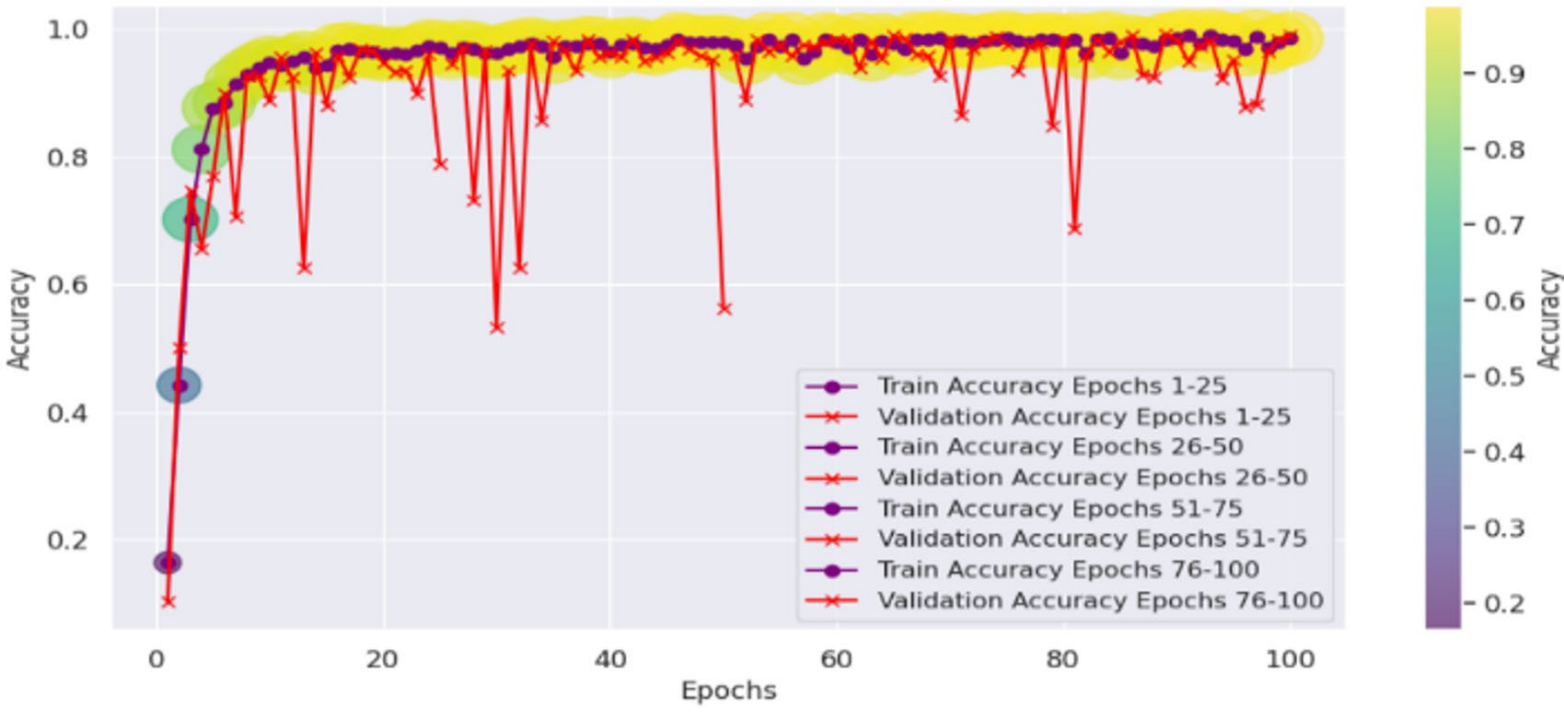
Accuracy Analysis of model over training and validation sets across epochs.

Furthermore, interpretation of the training and validation performance of the CvT model on the ASL Alphabet dataset helps one gain immense understanding about the convergence patterns, learning dynamics, and the generalization power of the proposed model over 100 epochs. The Fig. 26 depicting trends of training and validation accuracy during four groups of 25 epochs (epochs 1–25, 26–50, 51–75, and 76–100) emphasizes the gradual improvement of the training process. The general trend is that while the model continues improving as training progresses from Epochs 26 to 50, the CvT model becomes more stable, and training and validation accuracies converge to about or above 95%. And the last stages (Epochs 51–100) show the mature model because the training accuracy is more than 98% persistently, and the validation accuracy still sustains and drop less, suggesting better generalization.

**Fig. 27:**
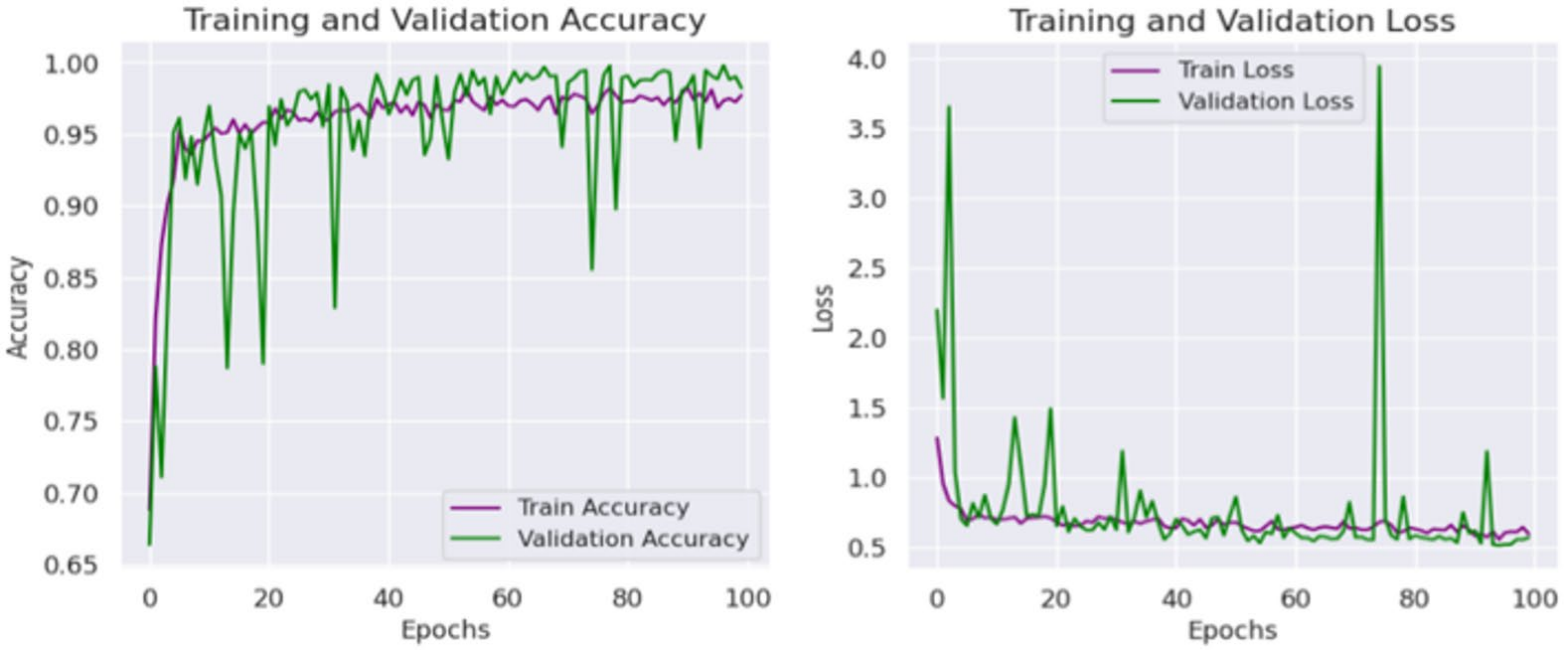
Accuracy and Loss Analysis of model based on ASL dataset.

Figure 27 shows the aggregated training and validation loss and accuracy curves. The left graph in turn confirms the prior analysis as the training and validation accuracy curves increase without signs of leveling off, reaching nearly 99% accuracy at the conclusion of training. The green accuracy validation curve just slightly lags the training curve, this was expected and it also shows that the model does not overfit too much. The training/validate loss curves also support this on the right. Both loss curves have considered early drop-offs, and with the training loss plateauing around 0.5 after only a few epochs. The validation loss, which is noisier due to small class imbalances and some overlapping of the classes, follows closely the training loss curve after the 30th epoch and lies low on average, confirming the capacity of generalization of the model on unseen data.

These visualizations together testify to the strength of the CvT model. They show that although there are some small fluctuations in validation due to diversity in the dataset, the model converges with an efficient solution to exhibit good accuracy and low loss, so it is effective for real-time SLR.

**Fig. 28:**
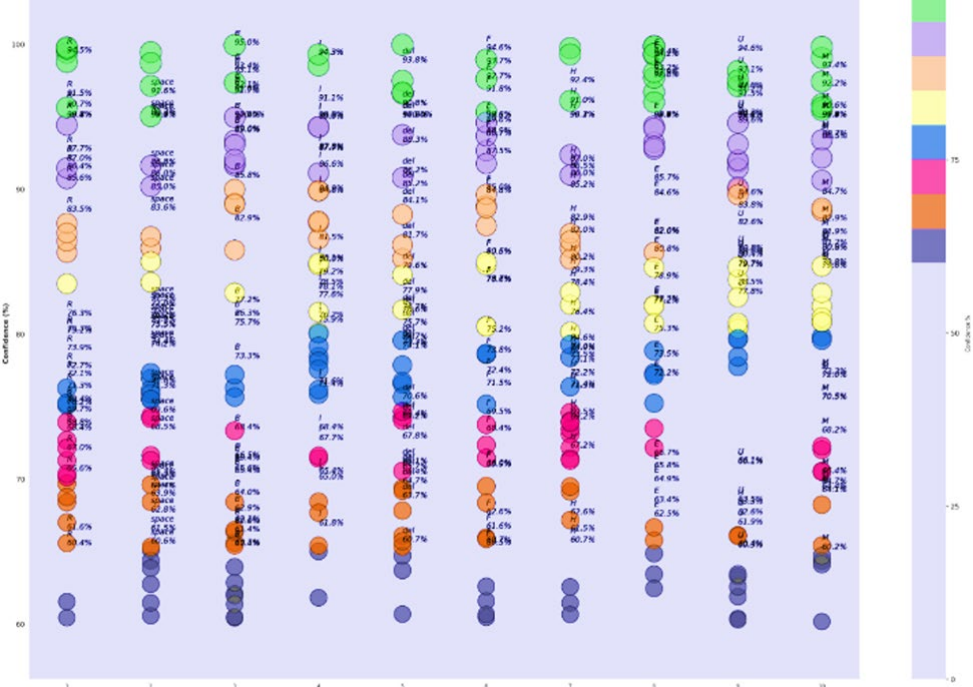
Confidence analysis based on test data for random prediction score.

The depicted confidence plot in Fig. 28 provides a statistical confidence-based measurement of performance of the proposed CvT model on the ASL Alphabet dataset. Each vertical column of predictions corresponds to one R, B, SPACE, DEL, etc., and the y-axis provides the confidence level (%) with which the model predicted each of the classes. Each image-associated bubble on the plot is an illustration of the decision based on a sample prediction, with bubble size indicating confidence strength and bubble color indicating a confidence percentage based on a scale of color. This plot shows most class predictions aggregating within a high-confidence range of 90% − 100%. Finally, also the ‘DEL’ and ‘SPACE’ gestures are indicative of good confidence, which means that the model successfully has learned patterns that are visually distinct and structurally consistent between these classes.

Nevertheless, there are a few classes for which the confidence scores are distributed more, such as the ‘R’, ‘M’, and ‘H’ ones, with posterior predictions varying from just above 90% to about 60%. ‘M’ for example has a higher variance with some samples scoring 60–70% confidence for ‘M’, possibly indicating intra-class variance (e.g., different users or subtle pose differences) or visual confusion with neighbors (‘N’ or ‘W’). These empirical distributions are crucial to know when and where the CvT model will require improvement or tuning, especially when data comes from real-world or uncontrolled conditions. From the perspective of statistical learning, the high-confidence predictions in most classes show that the CvT model can make robust learning and consistent recognition. The vertical distributions are sharp and far from the maximum value of 1, favoring light-tailed distributions that exhibit low dispersion and are well suited for deployment in assistive systems. The clustering in light green and yellow color tones for most predictions additionally supports that the model exhibits reliable confidence behavior over time. Overall, this confidence approach presents a statistical reinforcement of the CvT model, in addition to giving detailed interpretations on the class-wise certainty of the model. It also verifies that the model not only has the correct classification but also being sufficiently confident for most of the predictions. This visualization greatly assists in the fact that this model is highly capable for real-time application in SLR and provides some classes to be focused area for further improvement or adaptive training.

**Fig. 29:**
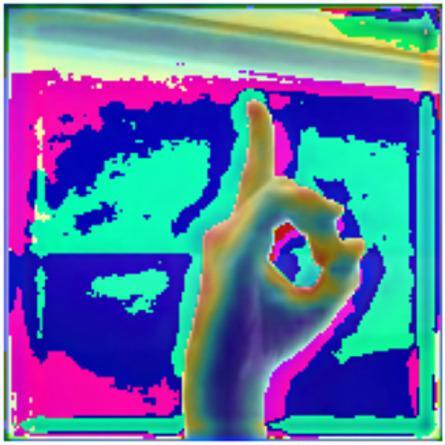
GRAD CAM interpretation depicts activation regions where the proposed model focuses during prediction, illustrating the relevance of visual features in decision-making.

Furthermore, for supporting the model performance, XAI performed using visualization of a Grad-CAM (Gradient-weighted Class Activation Mapping) heatmap from a SLR model, probably the CvT model, for classifying ASL (American Sign Language) gestures, as shown in Fig. 29. Grad-CAM is one of the most popular explainability techniques in deep learning, especially in computer vision, since it indicates the parts of the input image which contributed more to the decision making of the model.

The center part of the image is clearly emphasized between warm colors (yellow, red, or orange) and the hand making the gesture represents a strongly activated and important part in the decision process of the model. This indicates that the model’s convolutions and attention mechanisms were strongly tuned to the contours and shape of the fingers, especially the extended index finger and the circular form made by the thumb and index finger – two forms that have high semantic value for identifying individual ASL letters (‘F’ or ‘D’ depending on surrounding context).

The cooler colors (blue and cyan and, to a lesser extent, purple) are for the background, with some of that magenta-pink noise around the edges (which seems to have minimal effect on the model’s decision). This is a good property, as it suggests the model has learned to discard non-relevant background noise and focus on the gesture itself — an important characteristic for effective in-the-wild use in e.g. diverse/challenging or cluttered environments. This Grad-CAM interpretation indicates that the CvT model has spatial attention accuracy, as it properly discriminates and attends to relevant hand regions while omitting uninformative image regions. Gradient concentration along the finger edges and the palm boundaries show effective encoding of spatial features, likely due to CvT’s hybrid convolutional tokenization and transformer-based mechanism of applying global attention.

**Fig. 30:**
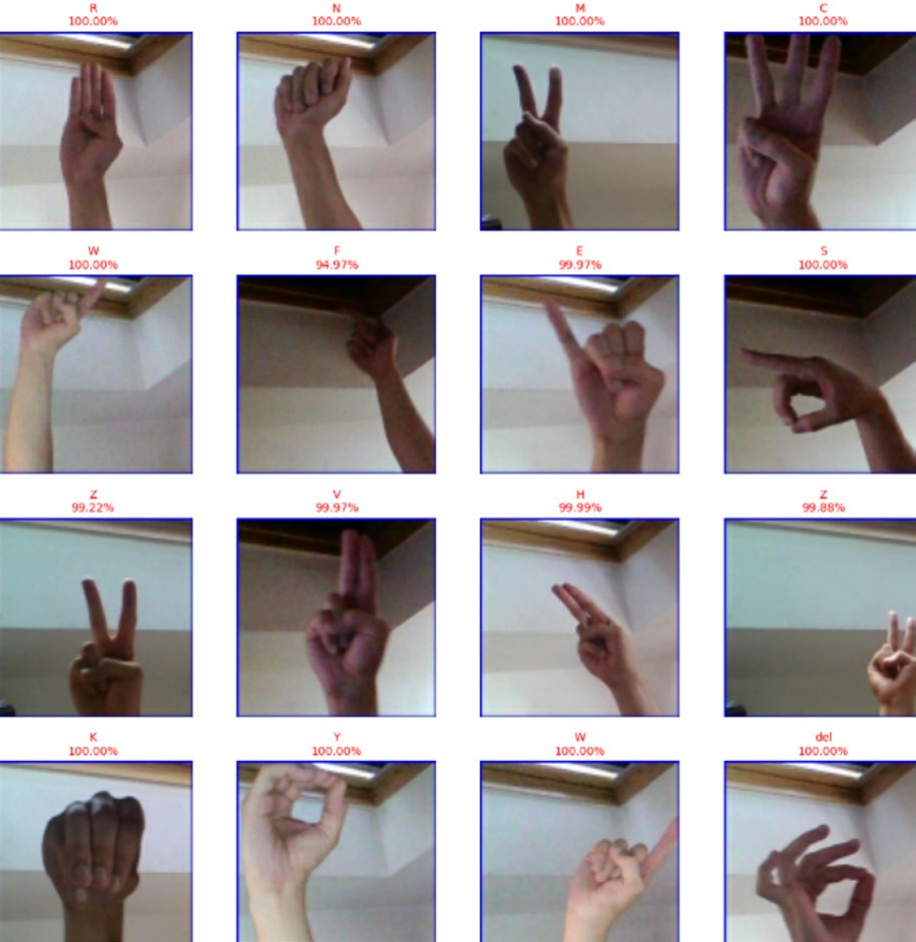
Predictive Results of Model showing class label prediction with ground truth performance analysis.

Furthermore, the clear detail and narrow location of the heatmap implies that the model has learnt precise internal representations of hand pose geometry, such as curvature, orientation, and articulation essential descriptors that are needed to differentiate between subtly differing instances of similar gestures. This visualization of Grad-CAM lends support to the efficiency and interpretability of the CvT model as it shows that it is capable to attend to the right visual cues. It prevents the model from making predictions based on spurious correlations (such as background textures or lighting artifacts) and enforces the mechanism of meaningful gesture semantics. This leads to high classification accuracy, but also the robustness of model, which is desirable for assistive technology and sign language interpretation systems in real time. The overall accuracy (99%) shows robust feature extraction and generalization based on predictive results are shown in Fig. 30.

### Comparison with existing studies

An improvement of 9% over the state of the art on transformer-based models in the literature is shown to be possible with the proposed CvT model to the task of SLR with 99% accuracy, as display in Table 12. Using standard transformer networks on an Isolated SLR Dataset, 89% accuracy is achieved by the 2020 study, significantly lower than CvT’s. This indicates that the early transformer models were not architected well enough to achieve robust SLR. Although the CNN + Vision Transformer hybrid approach from 2023 applied to Korean SLR datasets increased accuracy to 94%, the CNNs can be beneficial in spatial feature extraction.

**Table 12:** Existing results comparison with proposed model.

Ref	Models	Used Dataset	Results (Acc %)
^19^	CNN	ISL	89
^28^	CNN + ViT	Korean SLR	94
^25^	ViT	Arabic SLR	97
Proposed	**CvT**	**SLR**	**99**
		**ASL**	

**Fig. 31:**
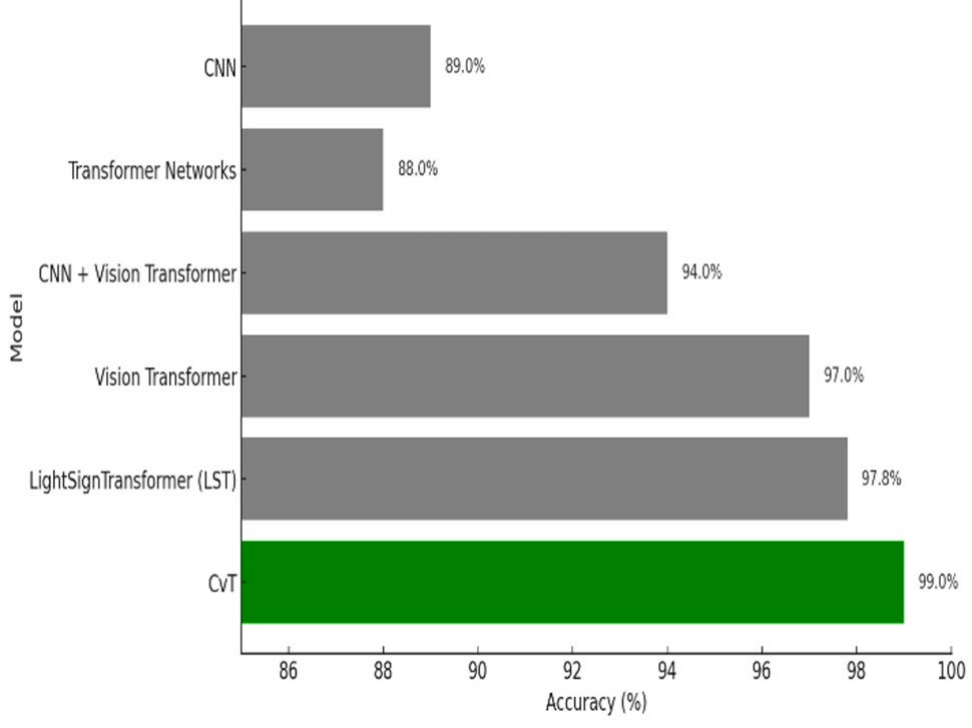
Comparative analysis of all applied models’ accuracy.

As before, transformer-based approaches have continued to improve their capability for recognition, to 97% accuracy with the 2024 Vision Transformer on Arabic SLR data. While this represents a step ahead of previous architectures, CvT outperforms the superset model for two possible reasons – it consistently outperforms with a simpler and more efficient method to keep local and global contextual dependency than standard vision transformers. However, CvT still achieves slightly higher accuracy (99%) due to its deep hierarchical tokenization strategy as well as deep hierarchical transformer layers for sign language feature extraction, as results comparison shown in Fig. 31. It compares vision transformers, hybrid models, showing that although significant progress. Such results show that CvT is the next step in advance in transformer-based SLR models.

## Conclusion

The findings demonstrate that proposed model significantly outperforms baseline models, including CNN and BeIT, achieving an outstanding accuracy of 99%. Compared to previous work, which achieved accuracy levels ranging from 88% to 97.8%, the model exhibits superior predictive capability, generalization strength, and confidence stability. The CvT model effectively mitigates misclassification issues observed in CNN and standard ViTs, particularly in visually similar sign gestures. Furthermore, confidence analysis validates the robustness of CvT, showing consistently high certainty across training, validation, and test datasets. These results underscore the potential of CvT for real-world deployment in assistive technologies and sign language translation systems. For future work, further optimizations can be explored, such as real-time deployment, multilingual sign recognition, and efficiency improvements for edge-device applications. Additionally, expanding the dataset to include custom real-based data and multimodal data covering dynamic gestures and contextual variations will enhance the model’s adaptability. This research paves the way for more advanced, AI-driven SLR systems, fostering inclusivity and bridging the communication gap for the deaf and hard-of-hearing communities worldwide especially in education sectors, communication and working pace.

## Data Availability

The datasets generated and/or analysed during the current study are available in the Kaggle repository: 1) https://www.kaggle.com/datasets/javaidahmadwani/sign-language-digits-dataset, 2) https://www.kaggle.com/datasets/grassknoted/asl-alphabet.

## Appendix

**Table:**

Symbol	Description
Terminology Used in Data Augmentation	
$\:\boldsymbol{x}$	Original input image
$\:{\boldsymbol{x}}^{\mathbf{{\prime\:}}}$	Preprocessed or augmented image
$\:\mathcal{R}(.)$	Resizing function
$\:\boldsymbol{h}\times\:\boldsymbol{w}\times\:\boldsymbol{c}$	Height, width, and number of image channels
$\:\mathcal{F}(\boldsymbol{x},\boldsymbol{p})$	Flipping function applied to input $\:x$
$\:\boldsymbol{p}\sim\:\boldsymbol{U}(0,1)$	Random horizontal-flip probability from uniform distribution
$\:\mathcal{T}(\boldsymbol{x},\boldsymbol{\theta\:})$	Rotation augmentation function
$\:\boldsymbol{\theta\:}\sim\:\boldsymbol{U}(-{20}^{\circ\:},{20}^{\circ\:})$	Random rotation angle sampled from uniform range
$\:\mathcal{G}(\boldsymbol{x},\boldsymbol{\sigma\:})$	Gaussian filtering function
$\:\boldsymbol{\sigma\:}=\frac{\mathrm{var}\left(\boldsymbol{x}\right)}{2}$	Variance-based dynamic filter width
$\:{\boldsymbol{x}}^{\boldsymbol{\gamma\:}}$	Gamma-corrected brightness enhanced image
$\:\boldsymbol{\gamma\:}\sim\:\boldsymbol{U}(0.8,1.2)$	Random gamma adjustment factor
Convolutional Token Embedding	
$\:{\mathcal{T}}_{\boldsymbol{c}}\left(\boldsymbol{x}\right)$	Convolutional token embedding function
$\:{\boldsymbol{W}}_{\boldsymbol{c}}(\boldsymbol{i},\boldsymbol{j})$	Trainable convolution filter weights
$\:{\boldsymbol{b}}_{\boldsymbol{c}}$	Bias term in convolutional projection
$\:\boldsymbol{k}$	Kernel size
$\:\boldsymbol{x}(\boldsymbol{i},\boldsymbol{j})$	Input feature value at spatial location $\:(i,j)$
Hierarchical Downsampling	
$\:\mathcal{D}\left({\boldsymbol{z}}_{\boldsymbol{l}}\right)$	Downsampling operator
$\:\boldsymbol{s}$	Downsampling stride
$\:{\boldsymbol{W}}_{\boldsymbol{D}}(\boldsymbol{m},\boldsymbol{n})$	Learnable convolutional downsampling filters
$\:{\boldsymbol{b}}_{\boldsymbol{D}}$	Bias used in downsampling stage
$\:\mathrm{ReLU}(\cdot\:)$	Activation function
Multi-Head Self-Attention	
$\:\mathrm{MSA}(\boldsymbol{Q},\boldsymbol{K},\boldsymbol{V})$	Multi-head self-attention mechanism
$\:\boldsymbol{H}$	Number of attention heads
$\:{\boldsymbol{\alpha\:}}_{\boldsymbol{h}}$	Learnable scaling factor for each head
$\:{\boldsymbol{Q}}_{\boldsymbol{h}},{\boldsymbol{K}}_{\boldsymbol{h}},{\boldsymbol{V}}_{\boldsymbol{h}}$	Query, Key, and Value matrices for head $\:h$
$\:{\boldsymbol{W}}_{\boldsymbol{Q}}^{\boldsymbol{h}},{\boldsymbol{W}}_{\boldsymbol{K}}^{\boldsymbol{h}},{\boldsymbol{W}}_{\boldsymbol{V}}^{\boldsymbol{h}}$	Trainable projection matrices for Q, K, V
$\:{\boldsymbol{d}}_{\boldsymbol{k}}$	Dimensionality of Keys used for scaled dot-product attention
$\:\mathrm{SoftMax}(\cdot\:)$	Normalized attention distribution
$\:\mathrm{FFN}\left(\boldsymbol{z}\right)$	Feedforward network applied on attended tokens
$\:{\boldsymbol{W}}_{1},{\boldsymbol{W}}_{2}$	Weight matrices of FFN layers
$\:{\boldsymbol{b}}_{1},{\boldsymbol{b}}_{2}$	Biases in FFN
$\:\boldsymbol{\sigma\:}\left(\boldsymbol{x}\right)$	Swish activation function $\:\frac{x}{1+{e}^{-x}}$
BeIT Masked Image Modeling Loss	
$\:{\mathcal{L}}_{\boldsymbol{B}\boldsymbol{e}\boldsymbol{I}\boldsymbol{T}}$	BeIT loss function for masked image modeling
$\:\boldsymbol{i}\in\:\mathcal{M}$	Index of masked input token
$\:\mathcal{M}$	Set of masked patches in the input image
$\:{\boldsymbol{\Phi\:}}_{\boldsymbol{\theta\:}}(\cdot\:)$	Decoder/prediction function parameterized by $\:\theta\:$
$\:{\boldsymbol{x}}_{\boldsymbol{i}}$	Ground-truth masked patch
$\:{\parallel\:\cdot\:\parallel\:}^{2}$	Squared L2 reconstruction error
